# Zinc Finger Transcription Factors Displaced SREBP Proteins as the Major Sterol Regulators during Saccharomycotina Evolution

**DOI:** 10.1371/journal.pgen.1004076

**Published:** 2014-01-16

**Authors:** Sarah L. Maguire, Can Wang, Linda M. Holland, François Brunel, Cécile Neuvéglise, Jean-Marc Nicaud, Martin Zavrel, Theodore C. White, Kenneth H. Wolfe, Geraldine Butler

**Affiliations:** 1 UCD School of Biomolecular and Biomedical Science, Conway Institute, University College Dublin, Belfield, Dublin, Ireland; 2 INRA UMR1319 Micalis, AgroParisTech, Jouy-en-Josas, France; 3 CNRS, Micalis, Jouy-en-Josas, France; 4 University of Missouri-Kansas City, School of Biological Sciences, Cell Biology and Biophysics, Kansas City, Missouri, United States of America; 5 UCD School of Medicine and Medical Science, Conway Institute, University College Dublin, Belfield, Dublin, Ireland; Vanderbilt University, United States of America

## Abstract

In most eukaryotes, including the majority of fungi, expression of sterol biosynthesis genes is regulated by Sterol-Regulatory Element Binding Proteins (SREBPs), which are basic helix-loop-helix transcription activators. However, in yeasts such as *Saccharomyces cerevisiae* and *Candida albicans* sterol synthesis is instead regulated by Upc2, an unrelated transcription factor with a Gal4-type zinc finger. The SREBPs in *S. cerevisiae* (Hms1) and *C. albicans* (Cph2) have lost a domain, are not major regulators of sterol synthesis, and instead regulate filamentous growth. We report here that rewiring of the sterol regulon, with Upc2 taking over from SREBP, likely occurred in the common ancestor of all Saccharomycotina. *Yarrowia lipolytica*, a deep-branching species, is the only genome known to contain intact and full-length orthologs of both SREBP (Sre1) and Upc2. Deleting *YlUPC2*, but not *YlSRE1*, confers susceptibility to azole drugs. Sterol levels are significantly reduced in the *YlUPC2* deletion. RNA-seq analysis shows that hypoxic regulation of sterol synthesis genes in *Y. lipolytica* is predominantly mediated by Upc2. However, YlSre1 still retains a role in hypoxic regulation; growth of *Y. lipolytica* in hypoxic conditions is reduced in a *Ylupc2* deletion and is abolished in a *Ylsre1/Ylupc2* double deletion, and YlSre1 regulates sterol gene expression during hypoxia adaptation. We show that *YlSRE1*, and to a lesser extent *YlUPC2*, are required for switching from yeast to filamentous growth in hypoxia. Sre1 appears to have an ancestral role in the regulation of filamentation, which became decoupled from its role in sterol gene regulation by the arrival of Upc2 in the Saccharomycotina.

## Author Summary

All but a few eukaryotes die without oxygen and respond dynamically to changes in the level of oxygen available to them. One ancient oxygen-requiring biochemical pathway in eukaryotes is the pathway for the biosynthesis of sterols, leading to cholesterol in animals and ergosterol in fungi. Mutations in this pathway are a frequent cause of azole drug resistance in pathogenic fungi. The regulatory mechanism for the sterol pathway is also widely conserved between animals and fungi and is centred on a transcription activator, SREBP, which forms part of a sterol-sensing complex. However, in one group of yeasts – the Saccharomycotina, which includes the major pathogen *Candida albicans* – control of the sterol pathway has been taken over by an unrelated regulatory protein, Upc2. We show here by analysis of the yeast *Yarrowia lipolytica* that the evolutionary switch from SREBP to Upc2 was a two-step process in which Upc2 appeared in an ancestor of Saccharomycotina, and SREBP subsequently lost its sterol-regulatory function while retaining an ancient role in filamentation.

## Introduction

Changes in gene regulatory networks are an important mechanism of evolutionary adaptation. Transcriptional re-wiring can result from gene loss, gene duplication, alterations in transcription factor binding sites, or changes in protein modularity that affect the interaction of transcription factors with other regulators [1], [2], [3], [4], [5]. A hybrid ancestral state may be resolved in different ways in different lineages, such as occurred in the regulation of cell type specific genes in the Saccharomycotina yeasts [1], [6] and the substitution of transcription factors regulating of ribosomal protein genes in Ascomycota fungi [2]. Other examples include substitution of the transcription factor Cph1 with Gal4 for regulation of galactose metabolism genes in the *Saccharomyces* clade [7], and changes in telomere binding proteins [8]. Large-scale analysis of promoter motifs and transcription factor conservation suggests that re-wiring of networks may be relatively common in eukaryotes [9]. However, most reported evolutionary changes involve the connection or disconnection of a group of target genes from a particular transcription factor, while the cellular function of the factor remains the same [9].

We describe here a major transcriptional re-wiring event that occurred in the evolution of sterol synthesis (an oxygen-dependent process) in eukaryotes. Regulation of sterol synthesis by Sterol Regulatory-element Binding Proteins (SREBPs) is very well conserved between metazoa and most fungi, but this conserved system has been disrupted in the clade of yeasts that includes *S. cerevisiae*. In these yeasts the role of SREBPs in sterol synthesis has been replaced by Upc2 (reviewed in [10], [11], [12]).

SREBPs regulate cholesterol synthesis and uptake, and fatty-acid synthesis in mammalian cells [13]. They are a family of transcription factors with a bHLH (basic Helix-loop-Helix) domain with a characteristic tyrosine residue. When sterol levels are high, SREBPs interact with the sterol-sensing protein Scap (SREBP cleavage-activating protein) and the complex is retained at the endoplasmic reticulum (ER) through association with INSIG (insulin-induced protein) [14]. When sterol levels drop, cholesterol no longer binds to Scap, disrupting the interaction with INSIG and resulting in transport of the SREBP-Scap complex to the Golgi apparatus [15]. Here, two proteases (site-1 protease and site-2 protease) cleave SREBP firstly in the loop within the Golgi lumen, and secondly to release the N-terminal domain. The N-terminus of SREBP enters the nucleus where it acts to regulate gene expression (reviewed in [10], [16]).

SREBPs are well conserved in many fungi and have been shown to regulate sterol synthesis in several species, particularly in response to low oxygen [10]. Not all components of the pathway are conserved. In Basidiomycetes (such as *Cryptococcus neoformans*) and in some Ascomycetes (e.g. *Schizosaccharomyces pombe*) SREBPs interact with Scap proteins, but the INSIG homolog appears to play no role in retention in the ER membrane [17]. The N-terminal region of SREBP is released by a single cleavage reaction. In *C. neoformans*, this cleavage is carried out by a homolog of the mammalian site-2 protease [18], [19]. In the Ascomycetes (*Sch. pombe* and *Aspergillus fumigatus*), processing does not require the site-1/site-2 proteases but instead uses the Dsc E3 ligase complex and the proteasome [20], [21], [22]. Some Ascomycete lineages such as Eurotiomycetes (including *A. fumigatus*) have lost Scap, and it is not clear what their sterol-sensing mechanism is [10], [11].

At least some fungal SREBPs are also regulated by oxygen levels independently of sterol levels. In *Sch. pombe*, oxygen-dependent degradation of the N-terminus of the SREBP protein (Sre1N) is regulated by Ofd1, a member of the prolyl hydroxylase family, and by Nro1, a nuclear protein [23], [24]. Ofd1 also regulates binding of Sre1N to sterol regulatory elements (SRE) [25].

Surprisingly, within the Saccharomycotina subphylum of the Ascomycetes, SREBP proteins appear to play little or no role in regulating oxygen-dependent expression of sterol biosynthesis genes. Some SREBP-like proteins, with the characteristic tyrosine in the bHLH domain, are present in these species, for example *C. albicans* Cph2 and *S. cerevisiae* Hms1 [10]. However, these proteins are often considerably shorter than their homologs in other Ascomycetes, and no role for them in sterol gene regulation has been demonstrated [26]. Instead, sterol gene expression in Saccharomycotina is controlled by Upc2 proteins, which have Gal4-type Zn_2_-Cys_6_ zinc finger domains, and are structurally unrelated to SREBPs. Members of the Upc2 family regulate expression of sterol and other hypoxic genes in *S. cerevisiae*, *Candida glabrata*, *C. albicans* and *C. parapsilosis* [27], [28], [29], [30], [31].

We observed that *Yarrowia lipolytica*, representing the most divergent known lineage of the Saccharomycotina, is unique among fungi in having a genome that contains readily identifiable genes for both SREBP and Upc2. It may thus be a ‘molecular fossil’ of a transition stage during the handover of control of sterol metabolism from SREBP to Upc2 in the Saccharomycotina. We show here that in *Y. lipolytica*, both Upc2 and SREBP play a role in responding to hypoxic conditions. However, Upc2 is the main regulator of sterol genes. SREBP (Sre1), and to a lesser extent Upc2, regulates filamentous growth. Our analysis suggests that re-wiring of the ergosterol synthesis module occurred in an early ancestor of the Saccharomycotina, when regulation was ceded to Upc2.

## Results

### Conservation of Sre1 and Upc2

**Figure 1:**
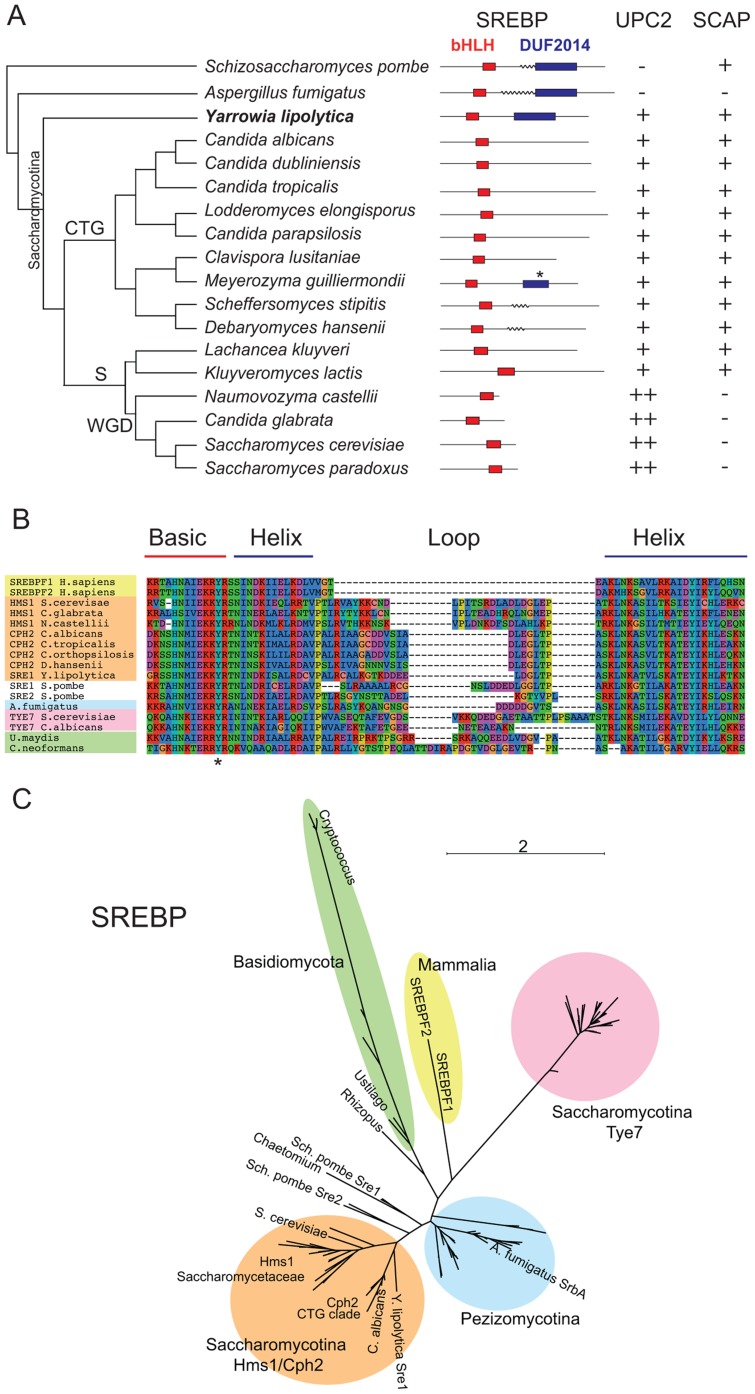
Conservation of sterol regulatory proteins in fungi. (A) Schematic phylogenetic tree of Saccharomycotina species and outgroups. Two domains from the SREBP proteins are shown diagrammatically as red (bHLH) and purple (DUF2014) boxes. Transmembrane domains are indicated by wavy lines; probabilities of <40% are not shown (details in Figure S1). The asterisk indicates a partially conserved DUF2014 domain in *M. guilliermondii*. The presence of Upc2-like proteins is indicated by + (one ortholog) or ++ (two orthologs). The presence/absence of Scap proteins is indicated with +/−. CTG = CTG clade, S = Saccharomycetaceae, WGD = Whole Genome Duplication clade. (B) Alignment of the bHLH domain from SREBP proteins from mammals (yellow), Basidiomycetes (green) and Ascomycetes (orange, blue, pink, white). The alignment was generated using MUSCLE implemented in SeaView [91]. The conserved Tyr residue is indicated with an asterisk. Clades are colored as in (C). (C) Unrooted phylogenetic tree of fungal SREBP-like proteins and mammalian SREBPF1/2. The tree was constructed from an alignment of bHLH regions (133 amino acid sites) by a maximum likelihood method. All sequences contain the atypical Tyr residue shown in (B). See Supplementary Figure S6 for taxon names and branch support values.

The Saccharomycotina is a lineage within the Ascomycetes that includes the Saccharomycetaceae and CTG clades (Figure 1A). The CTG clade contains *Candida* species in which the codon CTG is translated as serine rather than leucine [32], [33]. *Y. lipolytica*, a lipid degrading yeast [34], lies at the very base of the Saccharomycotina. *Y. lipolytica* has a gene (YALI0D15334) that is similar to Sre1 from *Sch. pombe* (maximum 30% identity) and SrbA from *A. fumigatus* (maximum 44%). One of the conserved regions corresponds to a bHLH domain near the N terminus, with a tyrosine rather than an arginine in the basic domain [35] (Figure 1A,B). The arginine-to-tyrosine substitution changes the DNA sequence that is bound, from the standard E-box recognized by most bHLH proteins to sequence called a sterol regulatory element-1 (SRE-1) [36]. The similarity to the SREBPs extends beyond the bHLH region. The *Y. lipolytica* protein also has a domain (DUF2014; Figure 1A) towards its C terminus that is shared with *Sch. pombe* Sre1 and *A. fumigatus* SrbA and other filamentous Ascomycetes. The function of this domain is unknown. It may be important for interaction with Scap and therefore for retention of SREBP in the membrane. However, SREBP proteins of *Aspergillus* species, which have lost Scap [10], retain the DUF2014 domain. The DUF2014 domain appears to have been acquired by the SREBPs in the ancestor of the Ascomycota, as it is not found in Sre1 of *C. neoformans* and other Basidiomycetes.

Most fungi have only one family of SREBP-like proteins, but *Saccharomyces* and *Candida* species have homologs of two potential SREBP-like protein families, both of which have bHLH domains with the characteristic tyrosine residue. One family (pink region in Figure 1C) is represented by Tye7 in *S. cerevisiae* and *C. albicans*. This family is restricted to the Saccharomycotina and is composed of short proteins (218–385 amino acids) without a DUF2014 domain. Tye7 regulates expression of glycolytic genes in *S. cerevisiae* and *C. albicans* [37], [38], [39], [40]. The second family (orange region in Figure 1C), is represented by Cph2 in *C. albicans* and Hms1 in *S. cerevisiae*. This family contains longer proteins with higher similarity to *Sch. pombe* Sre1 and Sre2, and has bHLH regions more similar to other SREBPs (Figure 1B,C). However, there is little conservation in the C-terminal regions between Hms1/Cph2 and the other fungal SREBPs, and the DUF2014 domain is not generally present, although in *Meyerozyma guilliermondii* (within the *Candida* clade) a remnant is recognizable (Pfam [41] E-value of 3.5e-4, compared to 3.4e-80 for the *Y. lipolytica* protein). Hms1 orthologs in *S. cerevisiae* and other species in the post-Whole Genome Duplication clade [42] are much smaller, and as a result the bHLH domain is closer to the C terminus (Figure 1A). The structure of the Hms1/Cph2 proteins including the apparent historical DUF2014 domain in *M. guilliermondii*, and their phylogenetic closeness to *Sch. pombe* Sre1 and the Pezizomycotina SREBPs, suggests that they (rather than Tye7) are the Saccharomycotina orthologs of SREBP. However, they have undergone substantial modification, including the degeneration of the DUF2014 domain. The *Y. lipolytica* protein, which we have named Sre1, is the only one within the Saccharomycotina species that retains an intact DUF2014 domain. It clearly falls within the Cph2/Hms1 clade (Figure 1C).

In *C. neoformans*, *Sch. pombe* and *A. fumigatus*, oxygen regulates the cleavage and localization of the SREBP transcription factor domain, releasing it from the ER and Golgi membrane and facilitating entry into the nucleus [18], [19], [20], [21], [22]. The *C. neoformans* and *Sch. pombe* proteins have two predicted membrane spanning domains, suggesting that both C and N termini are facing into the cytoplasm ([43], Figure 1A, Figure S1). SrbA from *A. fumigatus* is predicted to contain at least two (and possibly up to four) transmembrane domains, indicating that the protein is localized to membrane structures, with at least the N terminus facing the cytoplasm (Figure S1). Within the Saccharomycotina however there is very little evidence that the SREBP proteins are localized to membranes. For Sre1 from *Y. lipolytica* and Cph2 from *C. albicans*, there is <10% probability of one or two transmembrane domains respectively, and there is no indication of any transmembrane domain in the *S. cerevisiae* Hms1 protein.

**Figure 2:**
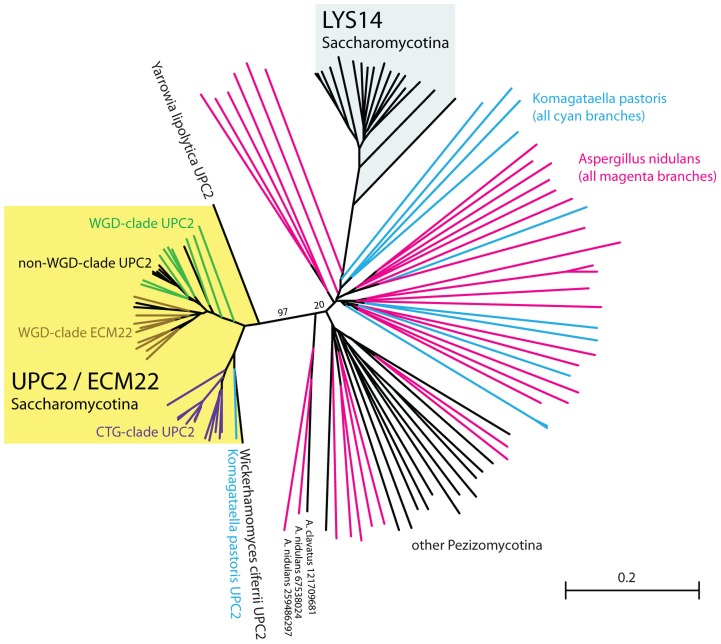
Upc2 has no clear orthologs outside the Saccharomycotina. This tree was constructed from sequences of Upc2 (and its ohnolog Ecm22 in WGD species); Lys14; and all Zn_2_Cys_6_ zinc finger protein genes in the genomes of two representative outgroup species, *Aspergillus nidulans* (magenta branches) and *Komagataella pastoris* (cyan branches) that have significant BLASTP hits (E<1e-6) when Upc2 proteins are used as a query. Also included are top-scoring BLASTP hits from other Pezizomycotina species (black branches). The tree was drawn by the neighbor-joining method without correction for multiple hits due to the high extent of sequence divergence. Bootstrap support for two key branches is indicated. NCBI gene identifier (gi) numbers are shown for the three sequences closest to the yellow Upc2/Ecm22 clade, but there is no evidence that these proteins are *Aspergillus* orthologs of Upc2. Scale bar represents 0.2 amino acid substitutions per site.

In contrast to SREBP, orthologs of Upc2 are only clearly identifiable within the Saccharomycotina (Figure 1A; Figure 2). In fact, in the post-Whole Genome Duplication clade, two paralogs (known as Upc2 and Ecm22 [30]) have been retained in all species (yellow clade in Figure 2). The Upc2 and Ecm22 proteins contain a fungal Zn_2_-Cys_6_ binuclear cluster domain, and a domain associated with fungal-specific transcription factors. The Upc2/Ecm22 proteins from the Saccharomycotina form a monophyletic clade that is not closely related to any other Zn_2_-Cys_6_ proteins of the Saccharomycotina (such as Lys14), or Ascomycota (Figure 2). The most likely interpretation of the phylogeny in Figure 2 is that Upc2 arose in the common ancestor of the Saccharomycotina, before the split between the *Y. lipolytica* lineage from the rest of the clade. Upc2 was presumably created by duplication of another zinc finger protein gene, but it has diverged to the point where its orthologs in species such as *Aspergillus* and *Komagataella*, if they exist, are unrecognizable (Figure 2).

### *Y. lipolytica* Upc2 regulates sterol metabolism

**Figure 3:**
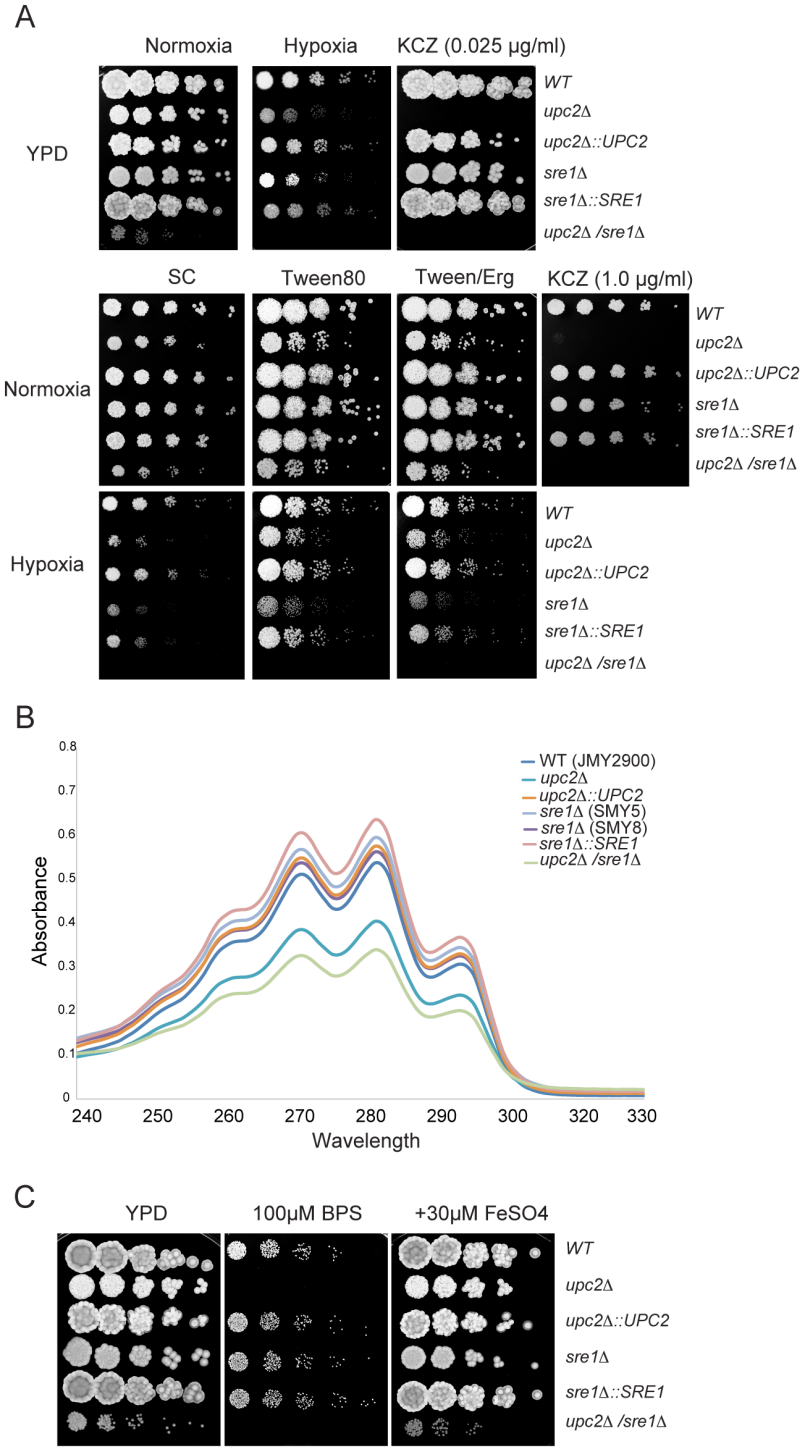
Role of YlUpc2 and YlSre1 in hypoxic growth, drug resistance and iron uptake. (A) *YlUPC2* controls susceptibility to azole drugs. *YlUPC2* and *YlSRE1* were deleted as described in Figure S2, and the deletion and re-constituted strains were plated as serial dilutions on YPD or synthetic complete (SC) media with additions as noted, and incubated in normoxic or hypoxic (1% O_2_) conditions. Deleting *YlUPC2* reduces growth in the presence of ketoconazole on YPD and SC media. Deleting *YlUPC2* or *YlSRE1* reduces growth in hypoxia, particularly on SC media. The strains shown are (in order): JMY2900, SMY2, SMY6, SMY5, SMY7 and SMY4. (B) Deleting *YlUPC2* reduces sterol content. The strains were grown in defined synthetic media and sterol levels were measured by absorbance as described in methods. The strains are the same as in (A), except that two independent deletions of *YlSRE1* were tested. The difference between the *Ylupc2* deletion and the wild type is statistically significant (P-value<0.05). (C) *YlUPC2* is required for iron uptake. The strains listed in (A) were spotted as serial dilutions on YPD, YPD +BPS and YPD+BPS+ additional iron.

To determine the role of the Sre1 (YALI0D15334) and Upc2 (YALI0B15818) orthologs in *Y. lipolytica* we knocked out both genes in the W29 background (Figure S2). *YlUPC2* was replaced with *URA3*, and *YlSRE1* with *LEU2*, in *Y. lipolytica* Po1d (*leu2–270, ura3–302* [44]) using fusion PCR [45] and previously described transformation methods [46]. The remaining markers (*LEU2* or *URA3*) were re-introduced into all strains, to reconstitute prototrophy (Table S1). *YlSRE1* and *YlUPC2* were also reintroduced at the endogenous locus by insertion of a cassette containing the relevant open reading frame plus 800 bp of the upstream region and a hygromycin resistance marker (HygEx). Deleting *YlSRE1* has a minimal effect on growth on rich media, whereas deleting *YlUPC2* reduces growth further, and the double deletion has a pronounced growth defect (Figure 3, growth curves are shown in Figure S3).

Deleting *YlUPC2* dramatically increases the susceptibility of *Y. lipolytica* to ketoconazole, whereas deleting *YlSRE1* has no obvious effect (Figure 3A). The level of drug required to inhibit growth is much higher when cells are grown on synthetic complete (SC) media than when grown on rich media (YPD); the reason for the difference is not known, but deleting *YUPC2* has the same effect on both media. The susceptibility phenotype is similar to that observed when *UPC2* is deleted in *S. cerevisiae* [47], *C. albicans* [48], [49] and *C. parapsilosis* [27], and when *SrbA* is deleted in *A. fumigatus* [50] and *SRE1* in *C. neoformans* [19]. Azole drugs target the ergosterol pathway in fungi, and in particular the product of the *ERG11* gene, which encodes Lanosterol 14-alpha-demethylase. We therefore measured the level of sterols in the various genetic backgrounds (Figure 3B). Cells were grown in defined minimal media, and sterols were extracted using an alcoholic KOH solution and heptane [49]. Figure 3B shows that deleting*YlUPC2* reduces absorbance at wavelengths that are indicative of lower sterol content, which are restored when the *YlUPC2* gene is re-introduced. Two independent deletions of *YlSRE1* had no reduction in sterol levels. There appears to be a slight additional reduction in the double deletion background relative to the *Ylupc2* deletion, but this is not statistically significant.

Deleting *YlUPC2* greatly reduces growth in hypoxic (1% O_2_) compared to normoxic conditions, during growth on both synthetic complete (SC) media containing methionine or rich (YPD) media (Figure 3A). Deleting *YlSRE1* also reduces hypoxic growth, which is more pronounced on defined media. The strain carrying deletions of both *YlUPC2* and *YlSRE1* grows poorly on YPD plates in normoxia, and fails to grow at all in hypoxic conditions. Adding fatty acids to SC media (in the form of Tween 80) improves growth of all strains, but the effect of deleting *YlUPC2* and *YLSRE1* is still evident (Figure 3). Addition of ergosterol does not rescue the phenotype any further, though this is possibly because *Y. lipolytica* cannot import sterols either aerobically or anaerobically (Figure S4). Reintroducing *YlUPC2* and *YlSRE1* in the single deletion strains restores growth in hypoxia (Figure 3).

In *Aspergillus fumigatus, SrbA* regulates expression of iron uptake genes, as well as of ergosterol synthesis [51]. We therefore tested the effect of deleting *YlUPC2* and *YlSRE1* on growth in low iron conditions. Figure 3C shows that when iron levels are depleted by adding the iron chelator BPS (4,7-diphenyl-1,10-phenanthrolinedisulfonic acid), the *Ylupc2* deletion strain fails to grow, whereas deleting *YlSRE1* has no effect. The phenotype is rescued by adding additional exogenous iron. In *Y. lipolytica*, *UPC2* therefore regulates both iron acquisition and sterol metabolism.

### Sre1 and Upc2 regulate filamentation in hypoxic conditions

**Figure 4:**
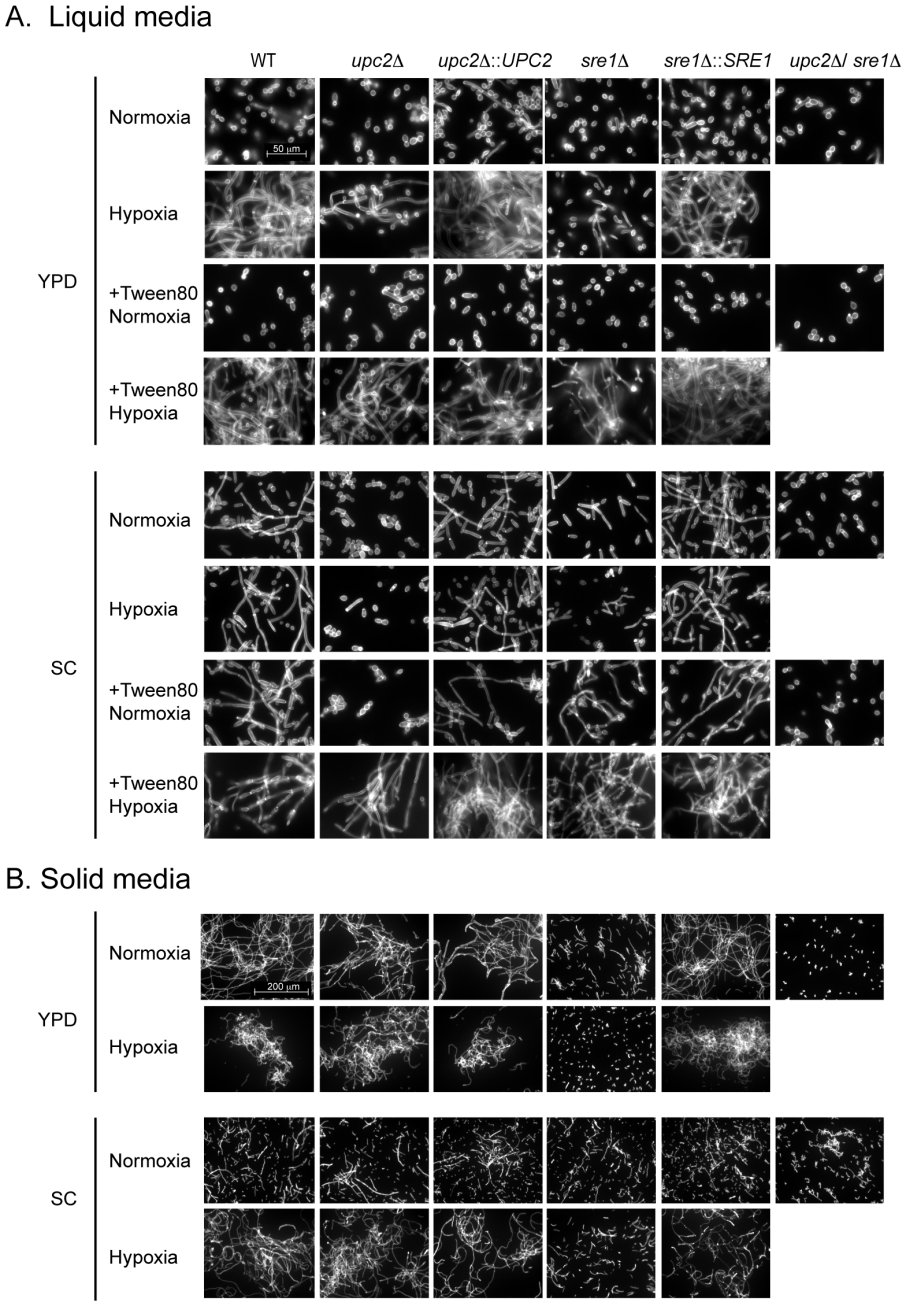
YlSre1 and YlUpc2 regulate filamentation. (A) Wild type (JMY2900), *Ylupc2* deletion (SMY2) and *UPC2* re-integration (SMY6), Ylsre1 deletion (SMY5) and *YlSRE1* re-integration (SMY7), and *ylupc2/Ylsre1* double deletion (SMY4) strains were grown overnight in liquid YPD or liquid SC media containing methionine, in normoxic or hypoxic (1% O_2_) conditions. Tween80 (1%) was added where indicated. The cells are stained with Calcofluor White. (B) The strains listed in (A) were grown on solid YPD or SC media in hypoxic or normoxic conditions for 2 days. Cells were removed from individual colonies, stained with Calcofluor White, and photographed.

*Y. lipolytica* and *C. albicans* are unusual among the Saccharomycotina species in that they can switch from growth as yeast cells to fully filamentous (hyphal) growth in certain conditions [52], [53]. Other species grow as yeast and pseudohyphae, or are locked in the filamentous form [54]. *Y. lipolytica* is truly dimorphic [34], [55]. Hyphae are induced by altering carbon source or pH, or by growing in hypoxic conditions [55], [56] [57]. We determined the effect of deleting *YlUPC2* and *YlSRE1* on hypoxia-induced filamentation of cells growing in rich (YPD) or minimal (SC) media, in both solid and liquid conditions (Figure 4). During growth in liquid YPD in normoxia, the strains are predominantly yeast-like, irrespective of the genetic background. Very few short filaments are formed. During growth in hypoxic conditions, the wild type and reconstituted strains are hyperfilamentous. The *Ylupc2* deletion also produces some long filaments. However, the *Ylsre1* deletion generates only very short filaments. Adding fatty acids (Tween 80) recovers the hypoxia-induced filamentation phenotype in the *Ylsre1* background, and improves filamentation of the *Ylupc2* deletion.

There is very little difference in cell morphology in cells growing in liquid SC in normoxic or hypoxic conditions. The wild type and reconstituted strains are filamentous, whereas the *Ylupc2* and *Ylsre1* deletions are predominantly yeast-like, with some short filaments found in the *Ylsre1* background. Adding Tween 80 rescues the induction of filamentation of *Ylupc2* and *Ylsre1* in hypoxic conditions, and partially increases filamentation of the *Ylsre1* deletion even in normoxia.

It has been reported that filamentation levels are higher on solid rather than liquid media [58]. On solid YPD media, we found that the wild type and the *Ylupc2* deletion strains are filamentous in both normoxic and hypoxic conditions. However, the *sre1* deletion fails to filament, even in hypoxia. On solid SC media, hypoxia induces filamentation of the wild type and the *Ylupc2* deletion, but not the *Ylsre1* deletion. Overall, our results show that YlSre1 is required for hypoxic-induced filamentation in all conditions and media tested, and YlUpc2 is required in most conditions. The double deletion remains in the yeast morphology, and fails to grow at all in hypoxia, suggesting that the two transcription factors act synergistically.

### Differential gene expression in hypoxia

To determine the roles of YlUpc2 and YlSre1 in regulating the hypoxic response, we first characterized the transcriptional profile of *Y. lipolytica* during growth in low oxygen. *Y. lipolytica* can tolerate oxygen levels as low as 1% (Figure 3), but it is incapable of anaerobic growth [52]. We used strand-specific RNA-seq to compare the transcriptional profile of cells grown in YPD in atmospheric oxygen levels and at 1% O_2_. Differentially expressed genes were identified using DESeq [59].

Approximately 1,900 genes are differentially expressed in low oxygen, corresponding to 30% of the genome (Table S2). This corresponds well with the response of *S. cerevisiae*, where >2,000 genes have altered expression in anaerobic conditions [60]. We used DAVID (Database for Annotation, Visualization and Integrated Discovery) to identify enrichment of specific categories among the differentially expressed genes [61]. DAVID applies several categorization tools, incorporating annotation categories that include Gene Ontology assignments, KEGG metabolic pathways, and InterPro database of protein families and domains. Genes upregulated in hypoxia are enriched for categories associated with membrane structure, ion binding and oxidoreductase activity (Table 1).

**Table 1:** Pathway enrichment of differentially expressed genes.

Category^a^	Term^b^	score^c^	Category^a^	Term^b^	score^c^
**Wild type in hypoxia/wild type in hypoxia**					
*Upregulated*			*Downregulated*		
Cluster 1	Intrinsic to membrane	7.75	Cluster 1	Ribosome	30.20
Cluster 2	Carboxypeptidase activity	3.09	Cluster 2	Ribosome biogenesis	2.98
Cluster 3	Ion binding	2.94	Cluster 3	ATP binding	2.30
Cluster 4	Zinc finger, C2H2	2.70	Cluster 4	Nucleotide binding	2.30
Cluster 5	Amino acid transport	2.60	Cluster 5	RNA binding	2.26
Cluster 6	Oxidoreductase activity	2.10	Cluster 6	Ribosomal protein	1.80
Cluster 7	Transcription factor activity	1.71	Cluster 7	DNA/RNA helicase	1.80
Cluster 8	Drug transport	1.63	Cluster 8	Translation	1.48
Cluster 9	Proteolysis	1.51	Cluster 9	WD repeat	1.44
Cluster 10	Signal peptide	1.37	Cluster 10	Microtubule function	1.41
Cluster 11	Nucleobase transmembrane transporter	1.37	Cluster 11	DNA-dependent ATPase	1.31
Cluster 12	Fatty acid biosynthesis	1.31			
***upc2*** ** deletion in normoxia/wild type in normoxia**					
*Upregulated*			*Downregulated*		
Cluster 1	Peptidase activity	3.10	Cluster 1	Steroid biosynthesis	2.57
Cluster 2	Exopeptidase	1.43			
***sre1*** ** deletion in normoxia/wild type in normoxia**					
*Upregulated*			*Downregulated*		
Cluster 1	Lipid metabolism	2.46	None		
Cluster 2	Thiolase	2.56			
Cluster 3	Fatty acid metabolism	2.07			
***upc2*** ** deletion in hypoxia/wild type in hypoxia**					
*Upregulated*			*Downregulated*		
Cluster 1	Proteolysis	2.93	Cluster 1	Ribosome	23.32
Cluster 2	Peroxisome	1.88	Cluster 2	Amino acid biosynthesis	2.10
Cluster 3	Glycerol metabolism	1.40	Cluster 3	Isocitrate dehydrogenase	1.93
			Cluster 4	Steroid biosynthesis	1.76
			Cluster 5	Cell redox	1.64
			Cluster 6	Antioxidant activity	1.59
			Cluster 7	Hydrogen transport	1.47
			Cluster 8	Glutathione-S-transferase	1.39
			Cluster 9	Fatty acid biosynthesis	1.33
***sre1*** ** deletion in hypoxia/wild type in hypoxia**					
*Upregulated*			*Downregulated*		
Cluster 1	Protein catabolism	7.23	Cluster 1	Ribosome	8.38
Cluster 2	Proteolysis	5.01	Cluster 2	Ribosome biogenesis	6.50
Cluster 3	Proteasome	3.98	Cluster 3	rRNA binding	1.89
Cluster 4	Autophagy	3.98			
Cluster 5	Ubiquitin	1.94			
Cluster 6	Serine peptidase	1.77			
Cluster 7	Plasma membrane	1.74			
Cluster 8	Ubiquitin protein ligase	1.64			
Cluster 9	ATPase, AAA type	1.56			
Cluster 10	Peroxisome	1.53			
Cluster 11	Phox-like	1.48			
Cluster 12	Ras GTPase	1.43			

^a^ Analysis of enrichment of functional clusters performed using DAVID.

^b^ One representative term from each cluster is shown. The entire dataset is available in Table S6.

^c^ Only clusters with an enrichment score >1.3 (P-value<0.05) are shown.

Notably, transcription factors are also significantly over-represented in genes upregulated in hypoxia (Table 1, Table S6). Expression of 78 genes with potential transcription factor activity is upregulated, including both *YlUPC2* and *YlSRE1* (Table S6). Expression of *HOY1*, a homeobox gene required for hyphal development in *Y. lipolytica* [58], [62], is among the genes with the highest fold induction. Interestingly, the transcription factor with the greatest increase in expression in hypoxia (YALI0C03564g) encodes a protein of unknown function, with a bHLH domain (Table S3). However, this domain does not contain the atypical Tyr residue, and probably binds to an E-box sequence rather than an SRE-1 element.

Downregulated genes are enriched in processes including ribosome biogenesis, rRNA processing, translation and microtubule function (Table 1, Table S6). These changes reflect the fact that the strains are growing slowly in hypoxic conditions (Figure 3, [63]).

### Regulation of the hypoxic response

**Figure 5:**
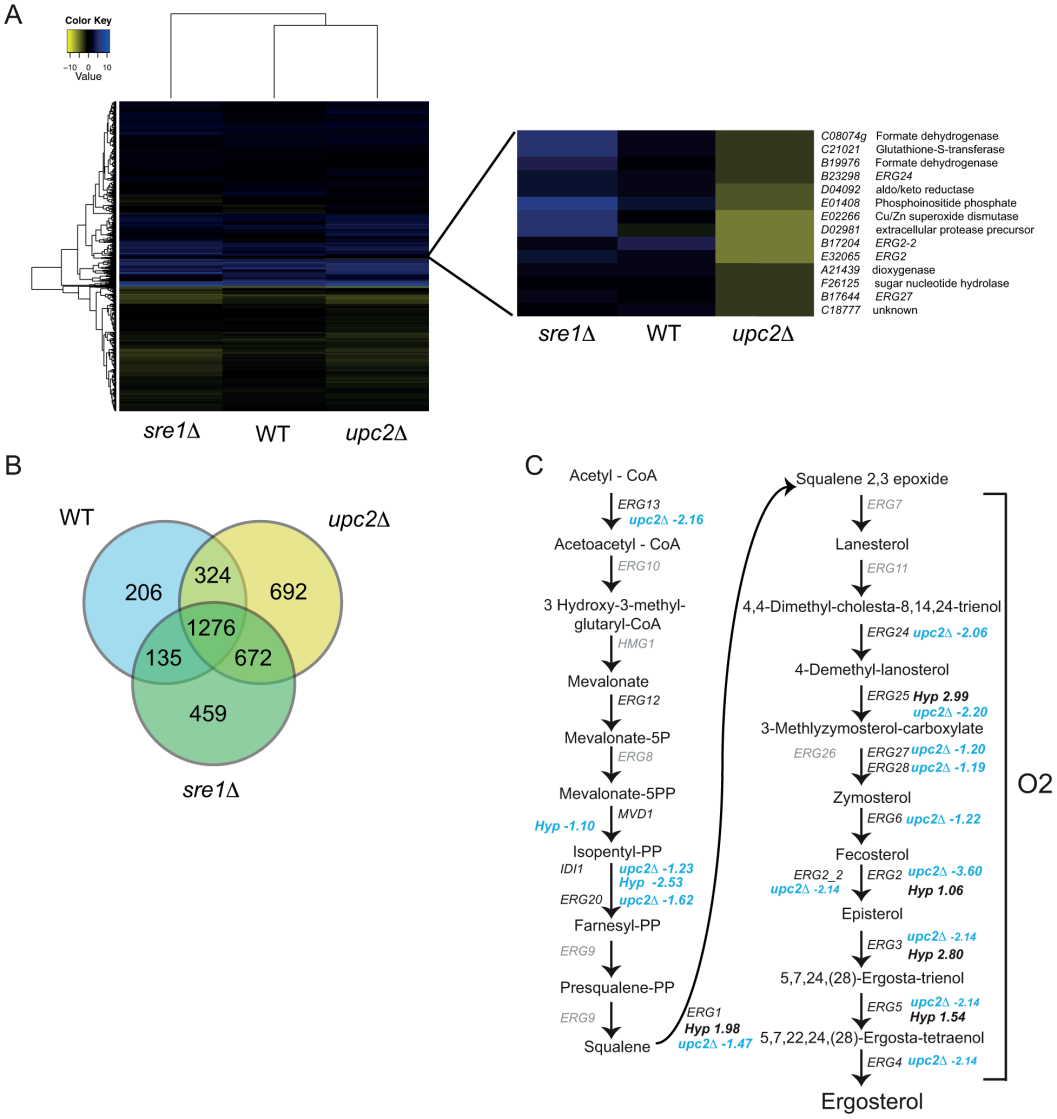
YlUpc2 regulates expression of ergosterol genes. (A) Hierarchical clustering of wild type (JMY2900), *Ylupc2Δ* (SMY2), and *Ylsre1Δ* (SMY5 and SMY8), all compared in hypoxia vs normoxia. Yellow indicates decreased expression in hypoxic growth. One cluster is shown in more detail. The full heatmap with gene names and descriptions is available in Figure S7. (B) Overlap in genes differentially expressed in hypoxia in wild type (JMY2900), *Ylupc2Δ* (SMY2) and *Ylsre1Δ* (SMY5 and SMY8) backgrounds. (C) Illustration of the sterol synthesis pathway in fungi. Changes in expression of indicated genes in wild type cells during growth in hypoxia compared to normoxia (Hyp) or in *Ylupc2* deletion strains compared to wild type cells both grown in hypoxia (*upc2Δ*) are indicated. Blue color indicates decreased expression and black color indicates increased expression. No changes in expression of genes in gray were identified.

To compare the effects of deleting *YlUPC2* and *YLSRE1* on the transcriptional profile of *Y. lipolytica* we analyzed the RNA-seq data using gene enrichment analysis implemented in DAVID [61], and by hierarchical clustering (Table 1, Figure 5, Figure S7). The overall transcriptional response of the deletion strains to hypoxia is very similar to the response of the wild type (Figure 5A,B). More than 1200 genes are differentially expressed in hypoxia in all three backgrounds (Figure 5B). This suggests that there are many other transcription factors apart from *UPC2* and *SRE1* that regulate the hypoxic response, supporting our analysis of hypoxic induction in wild type cells (Table 1).

Gene enrichment analysis shows that deleting *Ylupc2* results in lowered expression of steroid metabolism genes, even when the strains are grown in normoxic conditions (Table 1, Table S6). The effect of *YlUPC2* on sterol metabolism during hypoxic growth is also obvious from the hierarchical cluster analysis (Figure 5A). Expression of one cluster of 14 genes is notably reduced in the *Ylupc2* deletion, while remaining upregulated in the *Ylsre1* strain grown in hypoxia (Figure 5A). This group includes four genes required for ergosterol biosynthesis, all of which function in the oxygen-dependent part of the pathway. Two are paralogs of *ERG2* (C-8 sterol isomerase), one of which we have designated as *ERG2-2*. Expression of both is greatly reduced in the *Ylupc2* deletion, but not in the *Ylsre1* background. Most of the remaining genes in the cluster have roles in redox reactions, such as formate dehydrogenase, superoxide dismutase and glutathione-S-transferase.

**Figure 6:**
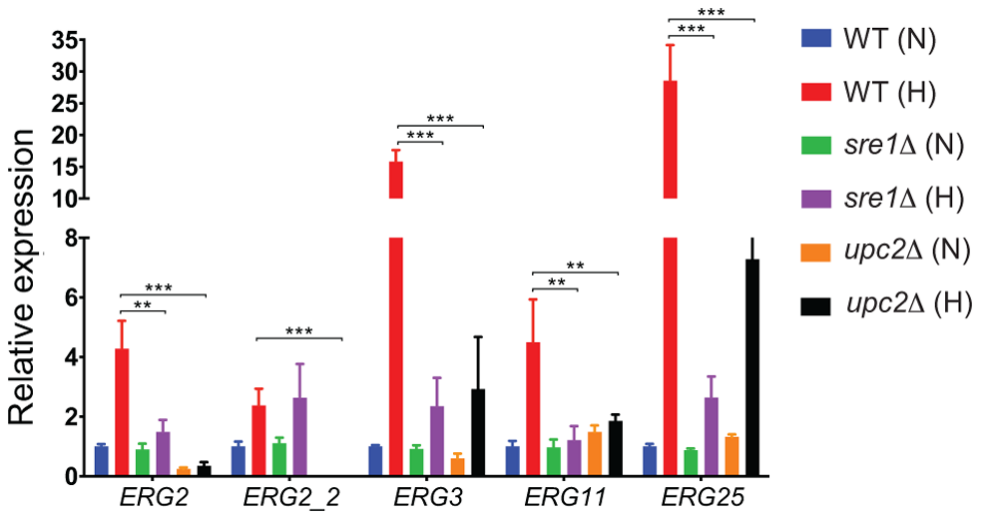
*YlUPC2* and *YlSRE1* regulate expression of ergosterol genes during hypoxic adaptation. Expression of the indicated *ERG* pathway genes was tested using qRT-PCR. All strains were grown to an A_600_ of 1.0 in SC media at 28°C under normoxic conditions (21% O_2_) and then switched to an hypoxic environment (1% O_2_) for 2 hours. Expression was normalized against actin, and is shown relative to the wild type strain grown in normoxia. Standard error of three replicates is shown. ** *P*<0.05, *** *P*<0.005.

Although not all of the ergosterol metabolism genes fall in the same cluster shown in Figure 5A, many are highly expressed in hypoxic conditions in wild type cells, and expression is greatly reduced (or abolished) in a *Ylupc2* deletion (Figure 5C). To determine if YlUpc2 is likely to be a direct regulator of *ERG* genes, we looked for evidence of enrichment of potential binding sites in the upstream promoters. We found that the Upc2 motif defined in *S. cerevisiae* is enriched in the promoter regions of ergosterol genes in all Saccharomycotina species, including *Y. lipolytica* (Table 2, genes shown in Figure 5C). In contrast, there is no enrichment in the equivalent promoters of *Sch. pombe* or *A. fumigatus*, species in which ergosterol genes are regulated by SREBPs [10]. Potential binding motifs were identified in 16 of the 21 promoters tested. *ERG2* and *ERG2-2*, the two genes with the strongest reduction in expression in the *Ylupc2* deletion (Figure 5A, Figure 6), have four potential binding sites each.

**Table 2:** Enrichment of Upc2 binding motif in 21 ergosterol synthesis genes.

Species	No. genes with motif	p-value
*Yarrowia lipolytica*	16	1.04E-04
*Saccharomyces cerevisiae*	18	3.16E-07
*Candida albicans*	17	8.04E-04
*Candida dubliniensis*	19	1.06E-04
*Candida parapsilosis*	18	7.95E-05
*Candida tropicalis*	19	3.54E-07
*Clavispora lusitaniae*	17	6.75E-06
*Meyerozyma guilliermondii*	14	1.91E-02
*Scheffersomyces stipitis*	21	6.80E-06
*Debaryomyces hansenii*	18	1.96E-05
*Lodderomyces elongisporus*	19	2.27E-03
*Naumovozyma castellii*	13	7.04E-11
*Candida glabrata*	10	5.80E-08
*Lachancea kluyveri*	18	1.37E-06
*Kluyveromyces lactis*	21	1.23E-10
*Schizosaccharomyces pombe*	9	0.22
*Aspergillus fumigatus*	4	0.70

Genes with reduced expression in the *Ylupc2* deletion relative to wild type in hypoxic conditions are also enriched for processes associated with cell redox, antioxidant activity and glutathione-S-transferase (Table 1, Table S6). YlUpc2 may therefore be involved in protection from oxidative stress. Many of the other downregulated genes are enriched for processes associated with translation, such as ribosome biogenesis and rRNA processes (Table 1, Table S6). This most likely reflects the fact that deleting *Ylupc2* further reduces growth in hypoxia (Figure 3).

Sre1 does not play a major role in regulating expression of sterol genes during long term growth in hypoxia; expression of the *ERG* genes in the *Ylsre1* deletion is very similar to that of the wild type cells grown in hypoxic conditions (Figure 5A,B), and sterol levels are not reduced in a *Ylsre1* deletion (Figure 3C). Deleting *YlSRE1* results in increased expression of lipid metabolism and fatty acid metabolism genes (Table 1, Table S6). However, unlike in mammalian cells, Drosophila, and *Sch. pombe*, the potential targets are mostly associated with lipid degradation, rather than biosynthesis (Table S6, [13], [64], [65]). The most highly enriched processes among downregulated genes compared to wild type are associated with ribosomal biogenesis and rRNA binding, which correlates with poor growth. Genes upregulated in the *Ylsre1* deletion relative to wild type are mostly associated with proteasome-dependent proteolysis, which may also result from slow growth (Table 2).

In the *Ylupc2/Ylsre1* double deletion, growth is greatly diminished and most downregulated genes are associated with translation (Table S6). Enrichment categories of upregulated genes are very similar to the categories upregulated during hypoxic growth of wild type cells (Table S6). It was not possible to determine the effect of deleting both *YlUPC2* and *YlSRE1* on the transcriptional response to hypoxia, because the double deletion strain fails to grow in low oxygen conditions (Figure 3).

For the RNA-seq experiments, cells were grown in rich media to minimize the effects of reduced cell growth, which is more pronounced in synthetic complete media (Figure 3). The strains were also grown for prolonged periods in hypoxic conditions. It is possible that the gene expression patterns would be different during growth in defined media, and during earlier stages of adaptation to low oxygen. We therefore used quantitative PCR to measure the expression of five genes in the ergosterol pathway in cells grown in synthetic complete media in high oxygen to mid log phase, which were shifted to hypoxic conditions for 2 hours. Figure 6 shows that *YlUPC2* is required for maximum hypoxic induction of all five genes. In particular, expression of *ERG2* and its paralog *ERG2-2* is completely dependent on *YlUPC2*, even in normoxic conditions. This pattern is similar to that observed in the RNA-seq experiments. However we find that *YlSRE1* is required for maximal hypoxic induction of at least four genes (*ERG2*, *ERG3*, *ERG11* and *ERG25*). It is therefore likely that YlSre1 plays a role in regulating expression of ergosterol genes at early stages of hypoxic adaptation.

## Discussion

### Upc2 regulates sterol synthesis in the Saccharomycotina

Sterols are essential for maintaining membrane structure and function, and synthesis in fungi and other eukaryotes is very carefully regulated at several levels [66]. Our results indicate that Upc2 is the major regulator of expression of sterol synthesis genes in *Y. lipolytica*. Expression of many of the sterol genes is reduced in the *Ylupc2* deletion, particularly during hypoxic growth, and the level of sterols in the cell is also reduced (Figure 3, Figure 5). Promoter analysis also indicates that the Upc2 binding sites are enriched in the promoters of sterol synthesis genes in *Y. lipolytica* and other species of the Saccharomycotina, but not in the equivalent promoters in *A. fumigatus* or *Sch. pombe* (Table 2). It is therefore likely that Upc2 homologs are the main regulators of sterol synthesis in all Saccharomycotina species. This has been shown experimentally for *C. albicans*, *C. parapsilosis*, *S. cerevisiae* and *C. glabrata* [27], [28], [29], [30], [31], [67], and now for *Y. lipolytica*.

Although the role of Upc2 is generally conserved, there are also substantial species-specific variations. Deleting *Ylupc2* results in a growth defect, which has not been reported in other Saccharomycotina species [30], [49]. YlUpc2 also regulates expression of *ERG* genes in normoxia (in particular, expression of *ERG2* and *ERG2-2*, Figure 6). In *C. albicans*, the role of Upc2 is generally only evident when ergosterol gene expression is induced with ketoconazole or growth in hypoxia, or with gain-of-function alleles of *UPC2* [29], [68]. In *S. cerevisiae*, Upc2/Ecm22 regulates expression of sterol synthesis and sterol uptake genes [30], [69]. The Upc2 paralogs control sterol import in *C. glabrata* [70], but not in *C. albicans* [31]. *Y. lipolytica* does not have an obvious ortholog of the *AUS1/PDR11* sterol transporters from *S. cerevisiae* [69], [71] nor of the regulator of sterol import, *SUT1* [72]. *Y. lipolytica* also apparently does not import cholesterol (and therefore probably ergosterol) in aerobic or hypoxic conditions (Figure S4).

**Figure 7:**
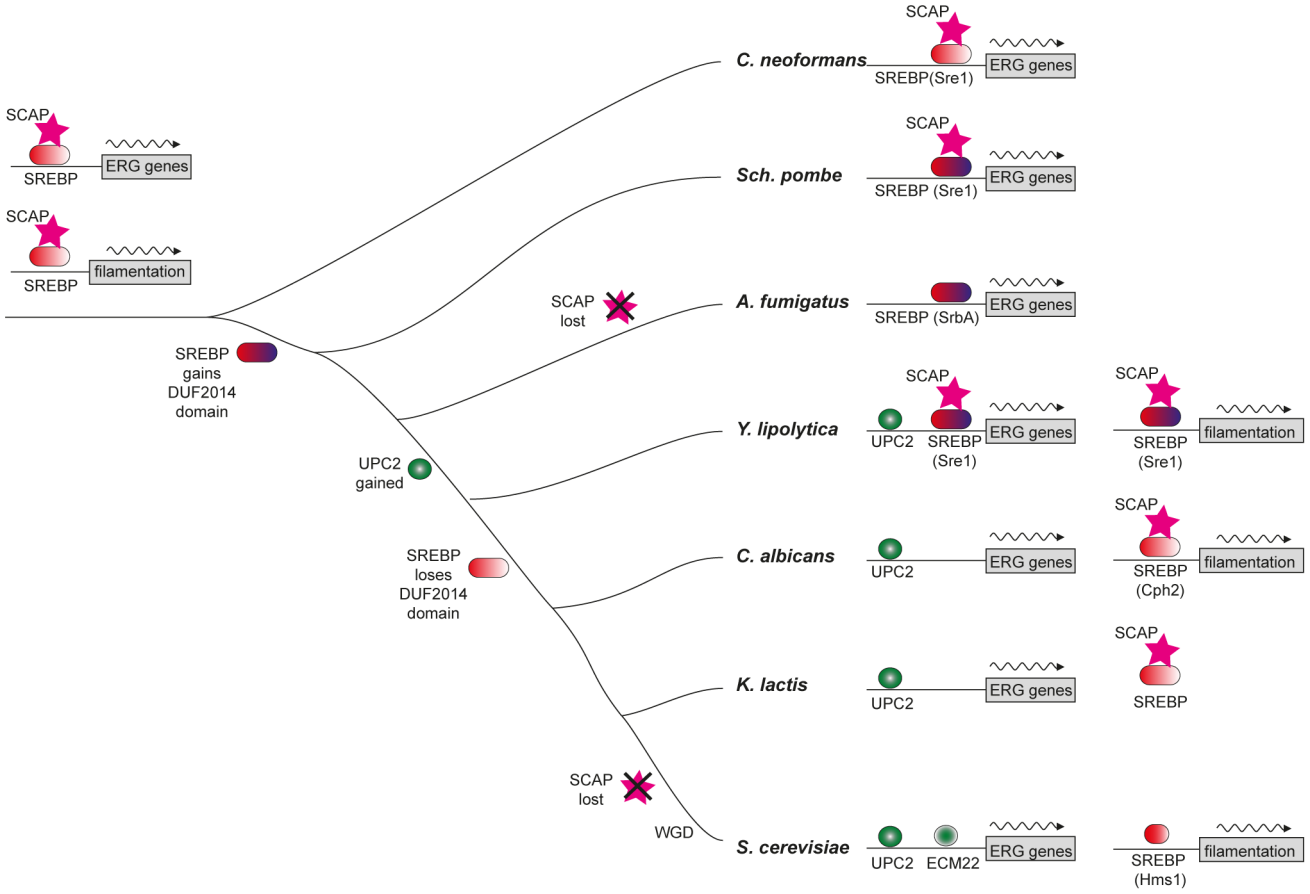
Model of sterol regulon evolution in Saccharomycotina. The hypothesized ancestral state, with sterol synthesis (ERG) genes and filamentation genes both being regulated by SREBP and Scap, is shown on the left. The DUF2014 domain of SREBP is shown in blue. SREBP replaced Upc2 in the promoters of ergosterol genes in the Saccharomycotina, but retains some role ergosterol metabolism in *Y. lipolytica*. Upc2 also regulates filamentation in *Y. lipolytica* (not shown). SREBP is known to have a role in regulation of filamentation in the three species shown. SREBP may have a similar function in *A. fumigatus*, where the deletion affects hyphal branching [50]. Loss of Scap is also indicated.

The Upc2 proteins in the Saccharomycotina are under considerable evolutionary constraint (indicated by short branch lengths in Figure 2), and are relatively distant from even their closest Pezizomycotina counterparts. This supports our hypothesis that Upc2 appeared in, or was substantially modified in, the ancestor of the Saccharomycotina (Figure 7), and the function has been generally conserved since. In *Y. lipolytica*, Upc2 also regulates iron uptake, which is controlled by SREBPs in *A. fumigatus* [51].

It is currently unknown how Upc2 proteins sense oxygen. However, the mechanism seems to be different from SREBP type of proteins. Our recent unpublished data indicate that Upc2 in *S. cerevisiae* does not undergo proteolytic cleavage, both its C- and N-terminus localize to the nucleus upon activation and its DNA binding domain seems to lose transcriptional activity without the protein's C-terminus. In addition, introducing an HA-tag at the C terminus of C. albicans Upc2 results in a gain-of-function, which may result from altered activation rather than processing [68].

### Role of Sre1 in the hypoxic response

SREBPs are major regulators of sterol synthesis and of the hypoxic response in Basidiomycete fungi (e.g. *C. neoformans*) and in some Ascomycetes (e.g. *Sch. pombe* and *A. fumigatus*, Figure 7) [10], [11], [12]. The SREBPs in the Ascomycetes gained a domain (DUF2014), whose function is currently unknown (Figure 7). DUF2014 is retained in the *Y. lipolytica* protein, but was lost in the most likely orthologs of Sre1 in the other Saccharomycotina species, including *S. cerevisiae* (Hms1) and *C. albicans* (Cph2). Hms1 and Cph2 have no known role in hypoxic regulation. However, we find that in *Y. lipolytica*, deleting *SRE1* reduces growth in hypoxic conditions (Figure 3). The defect in long-term hypoxic growth is unlikely to be due to regulation of sterol synthesis, because expression is not reduced in rich media (Figure 5A), and sterol levels are not reduced in the *Ylsre1* single deletion (Figure 3B). However, Sre1 contributes to induction of *ERG* genes during hypoxic adaptation (Figure 6). This suggests that whereas Upc2 is the major regulator of sterol genes, both Sre1 and Upc2 act synergistically at *ERG* promoters is some conditions (Figure 7). YlSre1 also has a role in response to long-term hypoxic growth that is separate from that of sterol biosynthesis.

The overall hypoxic response of *Y. lipolytica* is somewhat different to that of other fungi. Expression of fatty acid biosynthesis, drug transport and membrane proteins is increased, similar to that observed in fungi like *S. cerevisiae* and *C. albicans* (reviewed in [11]). Expression of sterol metabolism is also increased; although we do not find enrichment of sterol metabolism genes in the RNA-seq analysis using DAVID, it is clear from hierarchical clustering and from qRT-PCR (Figure 5, Figure 6). Unlike in *S. cerevisiae* and *C. albicans*, we did not observe changes in central carbon metabolism, such as upregulation of glycolysis and downregulation of the TCA cycle [29], [73]. Similar changes do occur in some obligate aerobes like *A. fumigatus* [74], but expression of glycolysis is reduced in others such as *Trichoderma reesii* [75]. In the aerobe *C. neoformans*, respiration is increased in low oxygen [19]. It is possible that we would observe different patterns in *Y. lipolytica* if we measured expression levels during short-term adaptation to hypoxia, or during growth in minimal media.

### Sre1 and Upc2 regulate filamentation

SREBP-like proteins from both *S. cerevisiae* and *C. albicans* are involved in regulating cell morphology. Overexpressing *HMS1* in *S. cerevisiae* results in hyperfilamentous growth [76]. The SREBP Cph2 from *C. albicans* was first isolated as a high copy inducer of pseudohyphal growth in *S. cerevisiae*, and it was subsequently shown that deleting *cph2* in *C. albicans* reduced the ability to switch to hyphal growth on Lees medium [26]. Hyphal induction in other conditions (such as growth following the addition of serum) is not impaired. Cph2 acts through the TEA/ATTS transcription factor *TEC1* to regulate filamentous growth. Several other pathways also regulate filamentation, including the Efg1-regulated cAMP-dependent protein kinase A (PKA) pathway, and the Cph1-mediated mitogen-activated protein kinase (MAPK) pathway (reviewed in [77]). The different pathways converge on some of the same target genes [78]. In *Y. lipolytica*, the equivalent MAPK pathway regulates filamentous (or mycelial) growth, whereas the cAMP-dependent PKA pathway is required for yeast-like growth [79], [80], [81], [82]. Filamentation is also regulated by Tec1; however, unlike *C. albicans*, Tec1 appears to promote yeast rather than hyphal growth [83].

We show that YlSre1 is required for hypoxia-induced filamentation, and that the filamentation phenotype is rescued by the addition of fatty acids (Figure 4). There are some differences in phenotype in liquid versus solid media and in rich versus minimal media, suggesting that there are several different signals inducing filamentous growth. However, deleting *YlSRE1* reduces filamentation in most conditions. Our results suggest that the bHLH Sre1-like proteins are ancient regulators of morphology, and indeed of filamentous growth, in the Saccharomycotina. Family members regulate cell morphology in at least three species – *S. cerevisiae*, *C. albicans*, and in the basal species *Y. lipolytica*. The role may be even older, as the Sre1 homolog in *A. fumigatus* (SrbA) is also required for cell polarity and hyphal branching [50]. We do not yet know how Sre1 regulates filamentation, though it may be linked to regulation of fatty acid synthesis. However, we note that one of the few known regulators of filamentation in *Y. lipolytica* (Hoy1 [58]) is induced in hypoxia (Tables S2, S3).

Upc2 also plays a role in hypoxia-induced filamentation in *Y. lipolytica* (Figure 4). The phenotype is generally less pronounced than deleting *Ylsre1*, especially in rich media. However, the double deletion fails to filament in any condition. It is therefore likely that in *Y. lipolytica*, Sre1 and Upc2 both regulate filamentation genes, similar to their dual role in regulating ergosterol metabolism.

In mammalian cells and in some fungi, SREBPs act together with sterol-sensing Scap proteins to regulate oxygen sensing [10]. Scaps retain SREBPs in the membrane in *C. neoformans* and *Sch. pombe*, though there is no homolog present in *A. fumigatus* or other Eurotiomycete species [10], [50]. There is a Scap homolog in *Y. lipolytica* (YALI0F00968p) that contains a sterol-sensing domain and several predicted transmembrane domains (Figure 7, Figure S5). There is also an apparent homolog in *C. albicans* and other species in the CTG clade, but these proteins have lost the sterol-sensing domain (Figure S5). Within the *Saccharomyces* clade, Scap has been lost from *S. cerevisiae* and its close relatives (Figure 7). However, Scap is present in other lineages, and a potential sterol-sensing domain is clearly identifiable (Figure 7, Figure S5). It therefore appears that Scap may play a role in sensing sterols (and therefore low oxygen) in some of the species in the Saccharomycotina. In other species (and in particular in the *Saccharomyces* and *Candida* clades) it is unlikely that Scaps and SREBPs are involved in sterol sensing.

We present here a significant example of transcriptional re-wiring, resulting from the substitution of SREBPs, conserved across a wide stretch of evolutionary time from Metazoa to fungi, by the Upc2 transcription factor that arose in the Saccharomycotina. The re-wiring is particularly important for fungal pathogens because expression of the sterol synthesis module confers susceptibility to azole drugs. We also describe a dramatic change in the function of SREBP homologs in the Saccharomycotina, from regulators of sterol synthesis to regulators of filamentation. Our results suggest that *Y. lipolytica* may represent a transitional stage, where both Upc2 and SREBPs contribute to regulation of sterol metabolism and filamentation.

## Materials and methods

### Media and strains

Yeast strains were maintained on solid YPD agar plates (1% yeast extract; 2% Bacto peptone; 2% glucose; 2% agar) at 28°C unless otherwise indicated. Hygromycin (Sigma) was added to YPD (1% yeast extra, 2% Bacto peptone, 2% glucose) at a final concentration of 300 µg/ml. Ketoconazole was added to YPD at a final concentration of 0.025 µg/ml and to SC at 1 µg/ml. Hypoxic conditions (1% O_2_, 99% N_2_) were obtained using an InVivo_2_ 400 hypoxic chamber. Transformants were selected on synthetic complete (SC) agar (0.19% yeast nitrogen base (YNB) without amino acids; 2% glucose; 2% agar; 0.5% ammonium sulfate; 0.075% amino acid drop-out mix lacking the relevant amino acid). For phenotype analysis, overnight cultures from single colonies were grown in liquid and solid SC (0.67% YNB, 2% glucose, 2% agar where required) or YPD media at 28°C and 200 rpm. 0.5 ml of the saturated overnight culture was washed twice with and resuspended in 1 ml PBS. Cells were diluted to 6.25×10^5^ cells in 1 ml PBS, and further 5-fold dilutions in PBS were generated. 3 µl were transferred to relevant media, incubated at 28°C at either 21% O_2_ or 1% O_2_ for 2 days and then photographed.

Bacterial strains were grown on LB agar without NaCl (1.5% agar; 1% tryptone; 0.5% yeast extract) supplemented with kanamycin at a final concentration of 50 µg/ml.

### Calcofluor White staining

For liquid cultures, cells from a single colony on SC medium were incubated in 10 ml YPD or SC media and 1% Tween 80- EtOH (vol∶vol = 1∶1) where indicated, and incubated at 28°C and 21% O_2_ or 1% O_2_ overnight. The cultures were washed, resuspended in PBS and 5 µl was mixed with 5 µl of 0.2 mM Calcofluor White (dissolved in 10 mM NaOH) and mounted on a glass slide with coverslip. Cells from colonies on solid media after 2 days growth were washed in PBS, resuspended in 100 µl PBS, and stained as above.

Cells were observed under UV fluorescence and photographed using a ColorView II camera mounted on a Zeiss AxioImager M1 fluorescent microscope using axiovision software.

### Gene deletions

Gene disruption cassettes were generated using fusion PCR (Figure S2). Approximately 1000 bp upstream from the start codon of *YlUPC2* and *YlSRE1* were amplified from *Y. lipolytica* Po1d using primers UPC2_p1 and UPC2_p2uraa or SRE1_p1 and SRE1_p2_leua/SRE1_p2_uraa, and from downstream of the stop codon using primers UPC2_t1_urab and UPC2_t2 or SRE1_t1_leub/SRE1_t1_urab and SRE1_t2 (Table S4). The *Y. lipolytica LEU2* and *URA3* genes were amplified from plasmids JMP802 and JMP803 [84], [85] using primers LEU-A and LEU-B or URA-A and URA-B. Primers UPC2_p2uraa and SRE1_p2_leua have 20 bp complementary to URA-A and LEU-A respectively (highlighted in bold, Table S4). Primers UPC2_t1urab and SRE1_t1_leub have 20 bp complementary to URA-B and LEU-B respectively (marked in bold, Table S4).

To make the complete disruption construct, the flanking regions and marker sequences were combined in a fusion PCR reaction using Ex Taq (TaKaRa Bio Inc.) with primers UPC2_p1 and UPC2_t2 or SRE1_p1 and SRE1_t2. The PCR conditions were 2 min at 94°, then 5 cycles of 30 s at 94°, 30 s at 60°, and 1.5 min at 72°, followed by 25 cyces of 30 s at 94°, 30 s at 60°, 3 min at 72°, and a final extension of 5 min at 72°. All PCR products were purified using a Qiagen PCR purification kit following the manufacturer's protocol and were introduced into *Y. lipolytica* by chemical transformation [46].

*UPC2* was replaced with *URA3* in *Y. lipolytica* Po1d generating strain SMY1. The *LEU2* marker was introduced into this background to make a prototrophic *upc2* deletion (SMY2). Similarly, *SRE1* was replaced with *LEU2* in *Y. lipolytica* Po1d generating strain SMY3. The *URA3* marker was introduced into this background to make a prototrophic *sre1* deletion (SMY8). *SRE1* was also replaced with *LEU2* in *Y. lipolytica* JMY330 (*URA3+*) generating a second prototrophic *Ylsre1* deletion strain, SMY5. To make the double deletion, *URA3* was used to replace *UPC2* in SMY3, generating the prototrophic strain SMY4. The *LEU2* marker was introduced into JMY330 to make a prototrophic version of *Y. lipolytica* Po1d (JMY2900) which is used as the wild type strain in this study.

To reintroduce *YlUPC2* and *YlSRE1*, regions from approximately 800 bp upstream of the start of the gene to the stop of the gene were amplified using primers URI_xhoI_F and URI_avrII_R or SRI_claI_F and SRI_bamHI_R. The forward primers introduce an *Xho*I (*YlUPC2*) or *Cla*I (*YlSRE1*) site and the reverse primers introduce *Avr*II or *Bam*HI sites. Digested products were cloned into plasmid JMP804 (unpublished) which contains a hygromycin (Hygex) resistance marker . Transformation was targeted to the *YlUPC2* or *YlSRE1* promoters by digestion with either *Psha*I or *Ppmu*I. The digested plasmids were introduced into SMY2 (*Ylupc2Δ*) or SMY5 (*Ylsre1Δ*) by chemical transformation [46] generating SMY6 (reconstituted *YlUPC2*) and SMY7 (reconstituted *YlSRE1*).

### Sterol measurements

All strains were grown in CSM complete medium (0.17% yeast nitrogen base without amino acids (Difco), 0.5% ammonium sulfate, 2% glucose, and supplemented with CSM supplement mixture (Sunrise Science Products)) for 48 h. Cultures equivalent to 100 A_600_ units were spun down and washed once with 10 ml of sterile water. The pellets were resuspended in 1 ml of an alcoholic KOH solution (12.5 g KOH, 17.5 ml H2O and filled to 50 ml with EtOH) and incubated at 85°C for 1 hour in 2 ml microfuge tubes. Finally 0.5 ml of heptane was added and vortexed for 3 minutes. After separation of the phases, the upper heptane phase was transferred to a new tube. Heptane extracts were diluted with 100% EtOH in 1∶5 ratio and absorbance between 230 and 320 nm was measured [49]. Three peaks of 270, 280 and 295 were used for quantification (T-test, p-value<0.05 for the *Ylupc2*/wild type comparison). The experiment was performed using three biological replicates, and the average measurements are presented.

### RNA-seq analysis

Cells were grown at 28°C overnight and then diluted to an A_600_ of 0.2, and grown until they reach an A_600_ of 1 at 28°C in YPD, in either 21% or 1% O2. Two to five biological replicates were used per sample (Table S5). Cells were harvested from 50 ml of culture by centrifugation, and either subjected to RNA extraction or frozen at −80°C. Total RNA was extracted from fresh or frozen cell pellets using a RiboPure Yeast Kit (Ambion). RNA concentrations were determined using a NanoDrop 1000 (Thermo Scientific), while quality and integrity was checked using a Bioanalyzer 2100 (Agilent Technologies). mRNA was prepared from total RNA using oligo dT Dynabeads (Invitrogen). 18 strand-specific libraries (Table S5) were generated by incorporation of dUTP as described in Guida et al [27], except that several samples were combined in one lane by multiplexing. One of 3 index adaptors (i6, i10 or i11, Table S4) was ligated to the samples to allow multiplexing. Adapters were ligated by mixing 25 µl of 2× Quick DNA Ligase Buffer (NEB), 1 µl (15 µM) of the specific adaptor mix, and 3 µl Quick T4 DNA Ligase with library samples. Ligations were carried out for 15 min at 20°C. The DNA was purified with a QIAquick PCR purification kit and MinElute column. The DNA was eluted with 10 µl EB.

Sequencing was carried out in-house on an Illumina Genome Analyzer IIx according to manufacturer's instructions, resulting in read lengths of approximately 42 bases. For four samples (Table S5) libraries were generated and sequenced by GATC using an Illumina HiSeq 2000. All data has been submitted to Gene Expression Omnibus and is available at accession number GSE47433.

### Read mapping and expression analysis

Gene annotations were obtained from Génolevures and manually curated using RNAseq data by the Neuvéglise group. In-house reads were processed according to version 1.8 of Illumina's Genome Analysis Pipeline. Multiplexed samples were separated using a Perl script and quality was tested using FASTQC (http://www.bioinformatics.babraham.ac.uk/projects/fastqc/). Each sample dataset was aligned to the 6 *Y. lipolytica* chromosomes using TopHat [86]. Reads mapped to two or more locations were removed from analysis. Data were visualized using the Artemis genome browser [87]. Raw counts of reads mapped to genes were calculated using HT-Seq. These were used as input for differential gene expression analysis using DESeq, with a P-value cutoff of < = 0.05 and LogFC cut off of > = 1 [59].

Differentially expressed gene lists were analyzed using the online DAVID functional annotation tool [61] with the *Y. lipolytica* gene background and default settings (Classification Stringency: Medium). Gene Ontology (GO) FAT terms, KEGG Pathways, InterPro and Swiss-Prot databases were selected for functional annotation clustering. Hierarchical cluster analysis implemented in R was used to identify genes with shared and different expression patterns in the wild type, *Ylsre1* deletion and Ylupc2 deletion when exposed to hypoxia. Genes that were differentially expressed in at least one comparison were clustered, using the log_2_ fold change values generated from DESeq analysis. Hierarchical cluster analysis implemented in R (http://www.R-project.org) was used to identify genes with shared and different expression patterns in the wild type, *Ylsre1* deletion and *Ylupc2* deletion when exposed to hypoxia. Genes that were differentially expressed in at least one comparison were included.

### qRT-PCR

Overnight cultures grown at 28°C in YPD media were washed twice with PBS and diluted to an A_600_ of 0.2 in SC media in two flasks. Cultures were grown to an A_600_ of 1.0 at 28°C in normoxic conditions (21% oxygen) and one flask was moved to a hypoxic environment (1% oxygen) for 2 hours. The normoxic sample was resuspended in RNAlater (Ambion) and frozen at −80°C. Total RNA was extracted and cDNA was prepared as described previously [88]. qRT-PCR was carried out on an Agilent Technologies Stratagene Mx2005p system using Brilliant III Ultra-Fast SYBR Green QPCR Master Mix (600882) as per the manufacturer's instructions. Two technical replicates were used for each sample. Cycling conditions consisted of 1 cycle at 95°C for 3 min followed by 40 cycles of 95°C for 10 s and 60°C for 30 s. A final cycle of 95°C for 1 min was followed by melting curve analysis performed at 55°C to 95°C (temperature transition, 0.2°C/sec) with stepwise fluorescence detection. Primers used for analysis are listed in Table S4. Relative expression changes were identified using the ΔCT method, compared to the expression of *ACT1*.

### Promoter motif analysis

The Upc2 binding site motif [89] was downloaded from the JASPAR database (http://jaspar.genereg.net/) [90] and used as input for tffind (http://globin.cse.psu.edu/dist/tffind/) to scan promoter regions from each genome (Table 2) using a cut off of 0.95 (95% confidence). A one-tailed Fisher exact test was performed to compare the enrichment among ergosterol pathway genes relative to the background group (rest of promoter regions in genome containing a motif). The number of binding sites per promoter was not considered. Fisher exact tests were calculated using the *R* Statistics package (http://www.r-project.org).

### Phylogenetic analysis

SREBP-like proteins were retrieved from the NCBI protein database using BLASTP with human SREBPF1, *Schizosaccharomyces pombe* Sre1, and *Cryptococcus neoformans* CNJ02310 as queries. Only proteins containing the atypical Tyr residue were retained. Sequences were imported into SeaView [91] for downstream analyses. Sequences from orthologous clades were aligned using MUSCLE [92], after which these clades were profile-aligned with each other using ClustalW2 [93]. Phylogenetic trees were constructed from the bHLH region of the alignment with PhyML, using the LG substitution model with four rate classes. Similar methods were used to construct phylogenetic trees for Upc2 and Scap proteins.

Transmembrane helices were predicted using the TMHMM server (http://www.cbs.dtu.dk/services/TMHMM/) [94] and protein domains were predicted using Pfam [41].

## Supporting Information

Figure S1 Analysis of transmembrane domains in SREBP proteins. Transmembrane domains were predicted using TMHMM [94]. The x-axes show the number of amino acids.
(PDF)

Figure S2 Knocking out *UPC2* and *SRE1* in *Y. lipolytica*. (A) Gene disruption cassettes were generated using fusion PCR. Regions approximately 1000 bp upstream from the 5′ end (P) of YlUPC2 and YlSRE1 were amplified from *Y. lipolytica* Po1d using primers 1 and 3 (UPC2_p1 and UPC2_p2uraa for *UPC2* and SRE1_p1 and SRE1_p2_leua for *SRE1*). Similarly, 1000 bp downstream from the 3′ end (T) of the genes were amplified using primers 4 and 6 (UPC2_t1_urab and UPC2_t2 or SRE1_t1_leub and SRE1_t2). The *Y. lipolytica LEU2* and *URA3* genes were amplified from plasmids JMP802 and JMP803 using primers 2 and 5 (LEU-A and LEU-B or URA-A and URA-B). Primers 2 and 3 and primers 4 and 5 have complementary ends. A fusion PCR cassette containing the marker gene and the upstream and downstream regions was generated by mixing the three fragments and amplifying with primers 1 and 6. The cassettes were transformed into *Y. lipolytica* Po1d using the lithium acetate method. (B) Disruption of the *YlUPC2* and *YlSRE1* genes was confirmed by PCR. Primers 7 and 8 (SRE1_out_F and SRE1_out_R or UPC2_out_F and UPC2_out_R) amplify a 4.5 kb fragment from *YlSRE1* in the wild type (JMY2900, Lane 1) and a 3.8 kb fragment from sre1::LEU2 (SMY3, Lane 2). Similarly, primers UPC2_out_F and UPC2_out_R amplify a 4.3 kb fragment from *YlUPC2* in the wild type strain (Lane 3) and a 3.6 kb fragment from upc2::URA3 (Lane 4, SMY2). A small band caused by non-specific PCR amplification is visible in some lanes (e.g. Lane 1). (C) Both *YlUPC2* and *YlSRE1* were restored by cloning the relevant open reading frame plus the promoter regions into plasmid JMP804 containing the hygromycin resistance marker HygEx. Integration was targeted to the upstream region of *YlUPC2* and *YlSRE1* by digestion with PshaI or PpmuI respectively. (D) The reintegrations were confirmed using primers 7 (UPC2_out_F for YlUPC2, SRE1_out_F for YlSRE1) and 9 (UPC2_in_R for YlUPC2, SRE1_in_R for YlSRE1). These amplify a 2 kb fragment from *YlSRE1* (wild type, JMY2900, lane 5) and the reconstituted sre1::LEU2::SRE1 (SMY7, lane 6). Similarly a 1.7 kb fragment is amplified from *YlUPC2* (wild type, JMY2900, lane 8) and from the reconstituted upc2:URA3::UPC2 strain, SMY6, lane 9). No bands are present in the deletion strains sre1::LEU2 (SMY5, lane 7) and upc2:URA3 (SMY2, lane 10).
(PDF)

Figure S3 Growth of wildtype (JMY2900), *Ylupc2* deletion (SMY2), *Ylsre1* deletion (SMY5), and double deletion (SMY4) strains in liquid YPD. The results show an average of three experiments. The standard deviations are very low and are not shown.
(PDF)

Figure S4 *Y. lipolytica* cannot import cholesterol. Sterol import on solid media was characterized by growth on YNB agar supplemented with fluorescently labeled cholesterol (0.25 µg/ml Cholesteryl BODIPY 542/563 (Invitrogen) in 1∶1 EtOH/Tween80). Overnight cultures were diluted to an A_600_ of 1.0, 3 µl were spotted on the agar plates and incubated for 48 hours at 28°C at 1% or 21% oxygen. Pictures were taken under normal light (A) or with a Typhoon 9410, Variable mode imager with excitation/emission of 532/555 nm (B). Sterol uptake is visualized by a zone of clearance around the colonies, as shown for the control *Candida glabrata* isolate. There are no clearance zones around the *Y. lipolytica* strains. The Ylsre1 deletion strain fails to filament in hypoxia, and there is little contrast under fluorescence conditions.
(PDF)

Figure S5 Scap protein evolution in fungi. (A) Transmembrane domains were predicted using TMHMM [94], and sterol-sensing domains using Pfam [41]. (B) The tree was constructed from full-length Scap sequences using PhyML. Black dots beside species names indicate proteins in which a sterol-sensing domain is predicted by PFAM. aLRT support values are shown. Numbers after species names are NCBI gi identifiers or CGOB gene names [95]. The Scap gene has been completely lost in the WGD clade of family Saccharomycetacae (including *S. cerevisiae*) but is present in all non-WGD Saccharomycetaceae such as *K. lactis* and *Z. rouxii*. The sterol-sensing domain of SCAP has been lost in all species of the CTG clade including *C. albicans*. The Scap gene is also missing from the Eurotiomycetes within the Pezizomycotina.
(PDF)

Figure S6 Details of the phylogenetic tree in Figure 1C. The tree has been rooted using the human SREBPF1/2 sequences. Protein sequences are identified by their NCBI gene identifier (gi) numbers except for sequences that were taken directly from the CGOB and YGOB databases [95]. Branch support values are aLRT (approximate likelihood ratio test) values from PhyML as implemented in SeaView [91].
(PDF)

Figure S7 Details of the hierarchical cluster shown in Figure 5A. Zoom for details.
(PDF)

Table S1 Strains used.
(DOCX)

Table S2 RNA-seq analysis of differentially expressed genes in *Y. lipolytica*.
(XLSX)

Table S3 Putative transcription factors upregulated in hypoxia.
(XLSX)

Table S4 Oligonucleotide primer sequences.
(DOCX)

Table S5 RNA-seq libraries.
(DOCX)

Table S6 Full list of enriched gene sets from Table 2.
(XLSX)
