# Supplementary material for: Zinc Finger Transcription Factors Displaced SREBP Proteins as the Major Sterol Regulators during Saccharomycotina Evolution
Source: PLoS Genet. 2014 Jan 16;10(1):e1004076. doi: 10.1371/journal.pgen.1004076 (PMC3894159; doi:10.1371/journal.pgen.1004076)
Supplement: Figure S1 — Analysis of transmembrane domains in SREBP proteins. Transmembrane domains were predicted using TMHMM [94]. The x-axes show the number of amino acids. (PDF) [file pgen.1004076.s001.pdf]

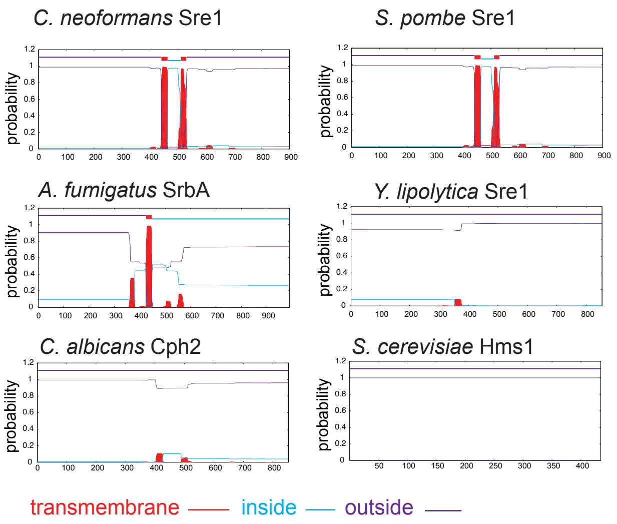

C. neoformans Sre1
S. pombe Sre1
A. fumigatus SrbA
Y. lipolytica Sre1
C. albicans Cph2
S. cerevisiae Hms1
probability
transmembrane
inside
outside
