# Supplementary figures and images for: Zinc Finger Transcription Factors Displaced SREBP Proteins as the Major Sterol Regulators during Saccharomycotina Evolution
Source: PLoS Genet. 2014 Jan 16;10(1):e1004076. doi: 10.1371/journal.pgen.1004076 (PMC3894159; doi:10.1371/journal.pgen.1004076)

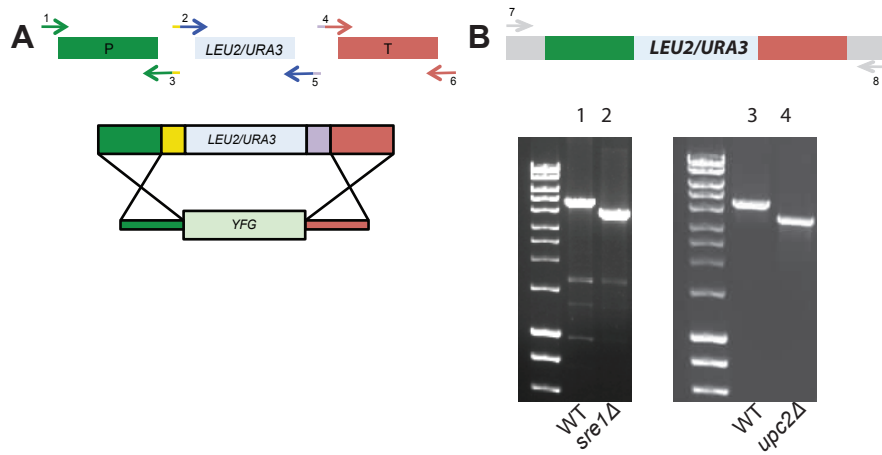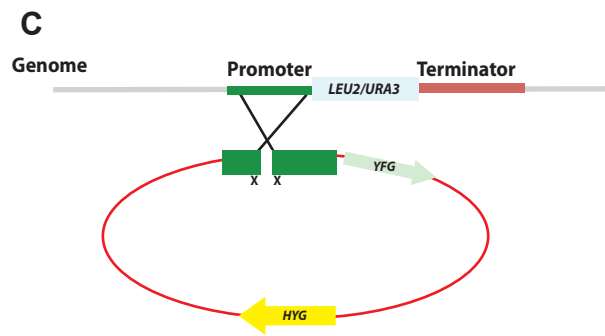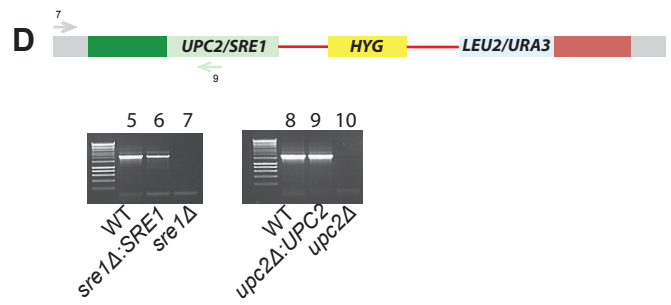

Supplement: Figure S2 — Knocking out UPC2 and SRE1 in Y. lipolytica. (A) Gene disruption cassettes were generated using fusion PCR. Regions approximately 1000 bp upstream from the 5′ end (P) of YlUPC2 and YlSRE1 were amplified from Y. lipolytica Po1d using primers 1 and 3 (UPC2_p1 and UPC2_p2uraa for UPC2 and SRE1_p1 and SRE1_p2_leua for SRE1). Similarly, 1000 bp downstream from the 3′ end (T) of the genes were amplified using primers 4 and 6 (UPC2_t1_urab and UPC2_t2 or SRE1_t1_leub and SRE1_t2). The Y. lipolytica LEU2 and URA3 genes were amplified from plasmids JMP802 and JMP803 using primers 2 and 5 (LEU-A and LEU-B or URA-A and URA-B). Primers 2 and 3 and primers 4 and 5 have complementary ends. A fusion PCR cassette containing the marker gene and the upstream and downstream regions was generated by mixing the three fragments and amplifying with primers 1 and 6. The cassettes were transformed into Y. lipolytica Po1d using the lithium acetate method. (B) Disruption of the YlUPC2 and YlSRE1 genes was confirmed by PCR. Primers 7 and 8 (SRE1_out_F and SRE1_out_R or UPC2_out_F and UPC2_out_R) amplify a 4.5 kb fragment from YlSRE1 in the wild type (JMY2900, Lane 1) and a 3.8 kb fragment from sre1::LEU2 (SMY3, Lane 2). Similarly, primers UPC2_out_F and UPC2_out_R amplify a 4.3 kb fragment from YlUPC2 in the wild type strain (Lane 3) and a 3.6 kb fragment from upc2::URA3 (Lane 4, SMY2). A small band caused by non-specific PCR amplification is visible in some lanes (e.g. Lane 1). (C) Both YlUPC2 and YlSRE1 were restored by cloning the relevant open reading frame plus the promoter regions into plasmid JMP804 containing the hygromycin resistance marker HygEx. Integration was targeted to the upstream region of YlUPC2 and YlSRE1 by digestion with PshaI or PpmuI respectively. (D) The reintegrations were confirmed using primers 7 (UPC2_out_F for YlUPC2, SRE1_out_F for YlSRE1) and 9 (UPC2_in_R for YlUPC2, SRE1_in_R for YlSRE1). These amplify a 2 kb fragment from YlSRE1 (wild type, JMY2900, lane 5) and [file pgen.1004076.s002.pdf]

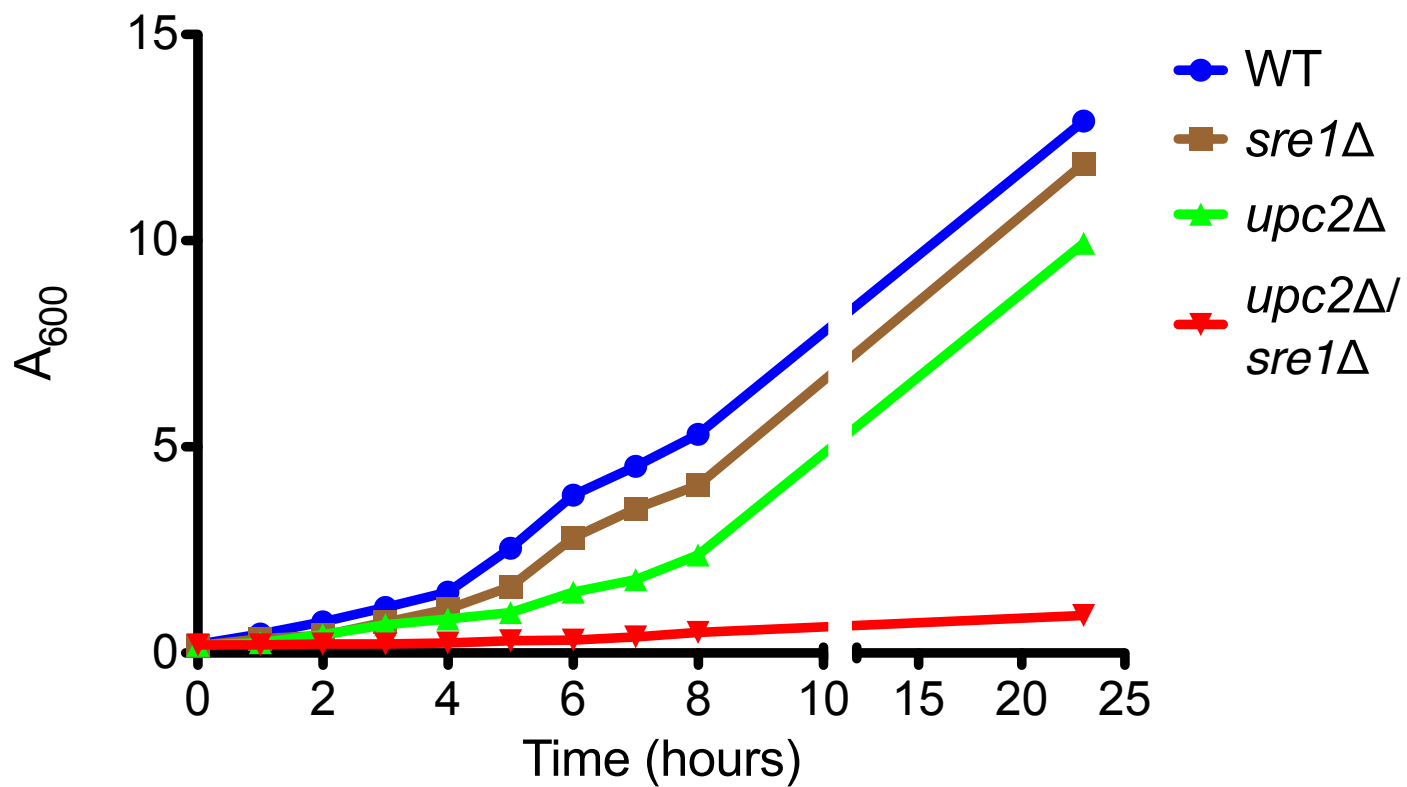

Supplement: Figure S3 — Growth of wildtype (JMY2900), Ylupc2 deletion (SMY2), Ylsre1 deletion (SMY5), and double deletion (SMY4) strains in liquid YPD. The results show an average of three experiments. The standard deviations are very low and are not shown. (PDF) [file pgen.1004076.s003.pdf]

A

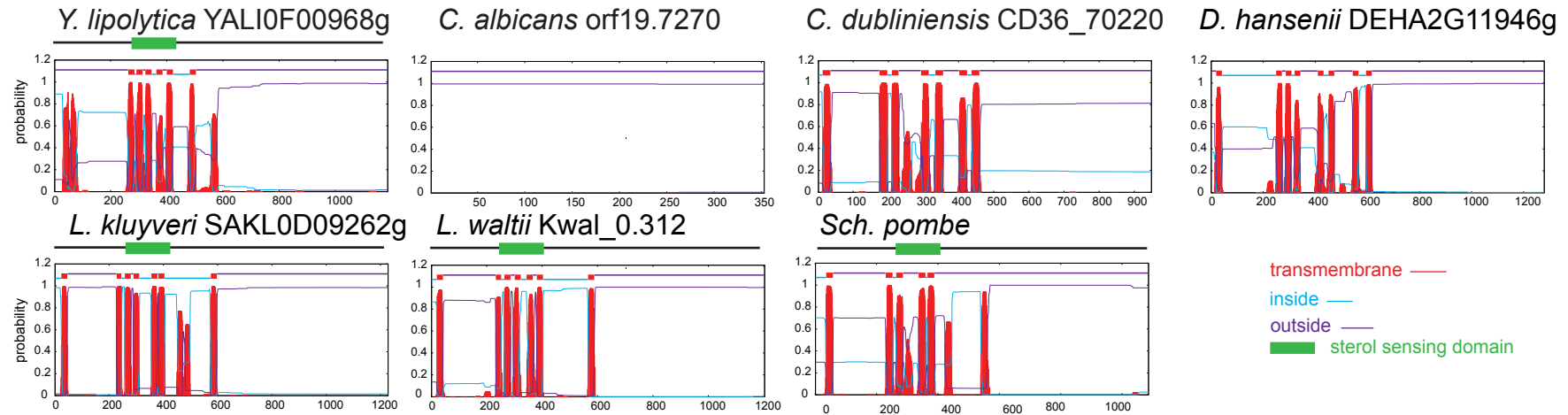

B

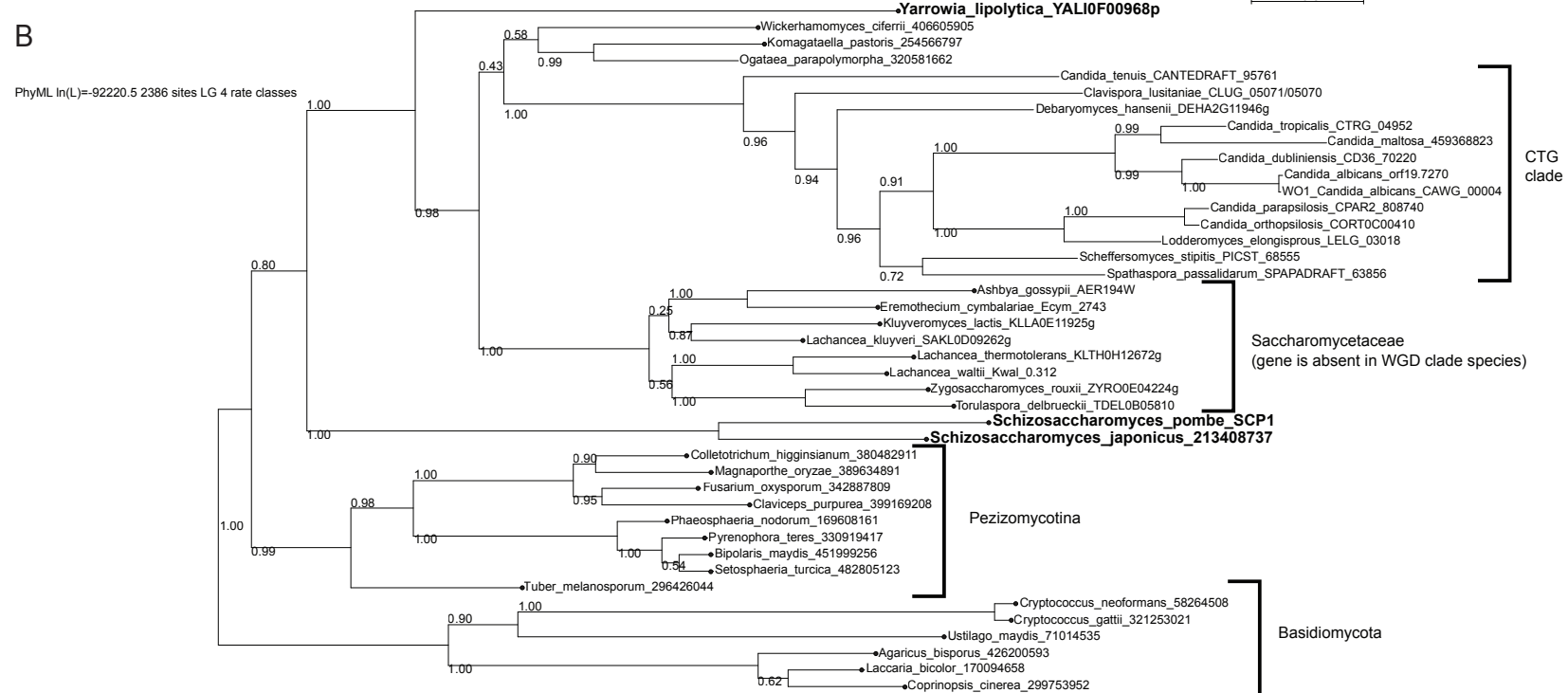

Supplement: Figure S5 — Scap protein evolution in fungi. (A) Transmembrane domains were predicted using TMHMM [94], and sterol-sensing domains using Pfam [41]. (B) The tree was constructed from full-length Scap sequences using PhyML. Black dots beside species names indicate proteins in which a sterol-sensing domain is predicted by PFAM. aLRT support values are shown. Numbers after species names are NCBI gi identifiers or CGOB gene names [95]. The Scap gene has been completely lost in the WGD clade of family Saccharomycetacae (including S. cerevisiae) but is present in all non-WGD Saccharomycetaceae such as K. lactis and Z. rouxii. The sterol-sensing domain of SCAP has been lost in all species of the CTG clade including C. albicans. The Scap gene is also missing from the Eurotiomycetes within the Pezizomycotina. (PDF) [file pgen.1004076.s005.pdf]

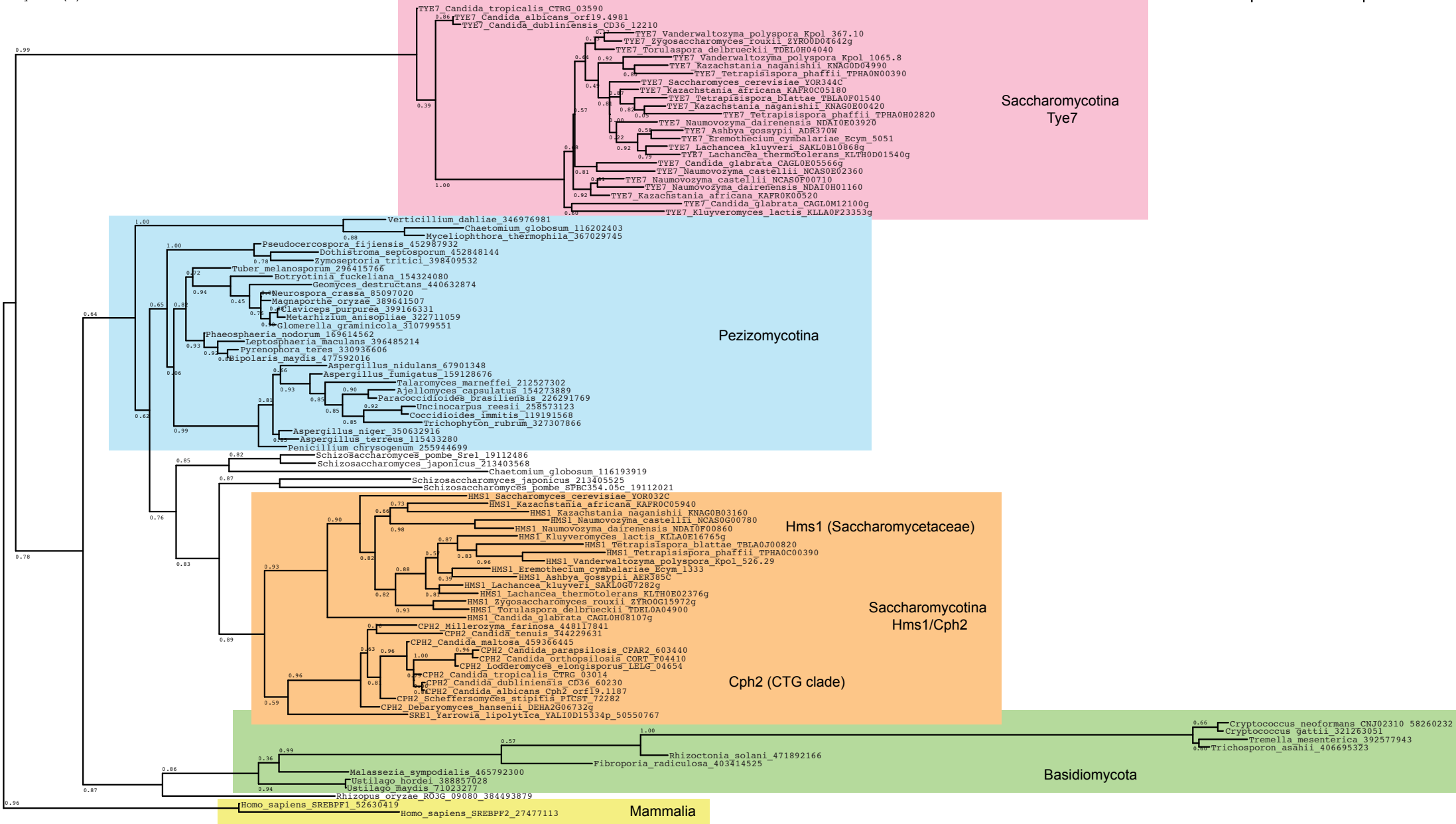

Supplement: Figure S6 — Details of the phylogenetic tree in Figure 1C. The tree has been rooted using the human SREBPF1/2 sequences. Protein sequences are identified by their NCBI gene identifier (gi) numbers except for sequences that were taken directly from the CGOB and YGOB databases [95]. Branch support values are aLRT (approximate likelihood ratio test) values from PhyML as implemented in SeaView [91]. (PDF) [file pgen.1004076.s006.pdf]

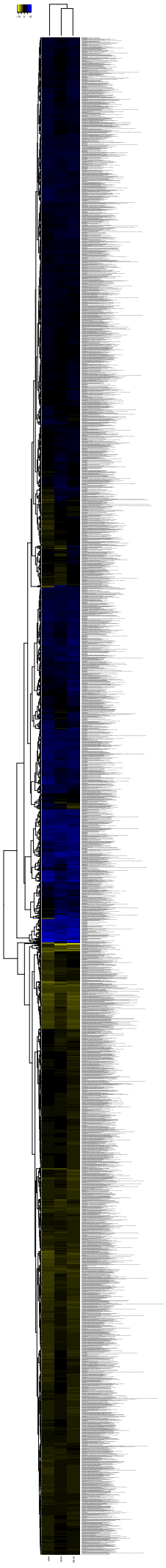

Supplement: Figure S7 — Details of the hierarchical cluster shown in Figure 5A. Zoom for details. (PDF) [file pgen.1004076.s007.pdf]
