# Supplementary material for: Zinc Finger Transcription Factors Displaced SREBP Proteins as the Major Sterol Regulators during Saccharomycotina Evolution
Source: PLoS Genet. 2014 Jan 16;10(1):e1004076. doi: 10.1371/journal.pgen.1004076 (PMC3894159; doi:10.1371/journal.pgen.1004076)
Supplement: Figure S4 — Y. lipolytica cannot import cholesterol. Sterol import on solid media was characterized by growth on YNB agar supplemented with fluorescently labeled cholesterol (0.25 µg/ml Cholesteryl BODIPY 542/563 (Invitrogen) in 1∶1 EtOH/Tween80). Overnight cultures were diluted to an A600 of 1.0, 3 µl were spotted on the agar plates and incubated for 48 hours at 28°C at 1% or 21% oxygen. Pictures were taken under normal light (A) or with a Typhoon 9410, Variable mode imager with excitation/emission of 532/555 nm (B). Sterol uptake is visualized by a zone of clearance around the colonies, as shown for the control Candida glabrata isolate. There are no clearance zones around the Y. lipolytica strains. The Ylsre1 deletion strain fails to filament in hypoxia, and there is little contrast under fluorescence conditions. (PDF) [file pgen.1004076.s004.pdf]

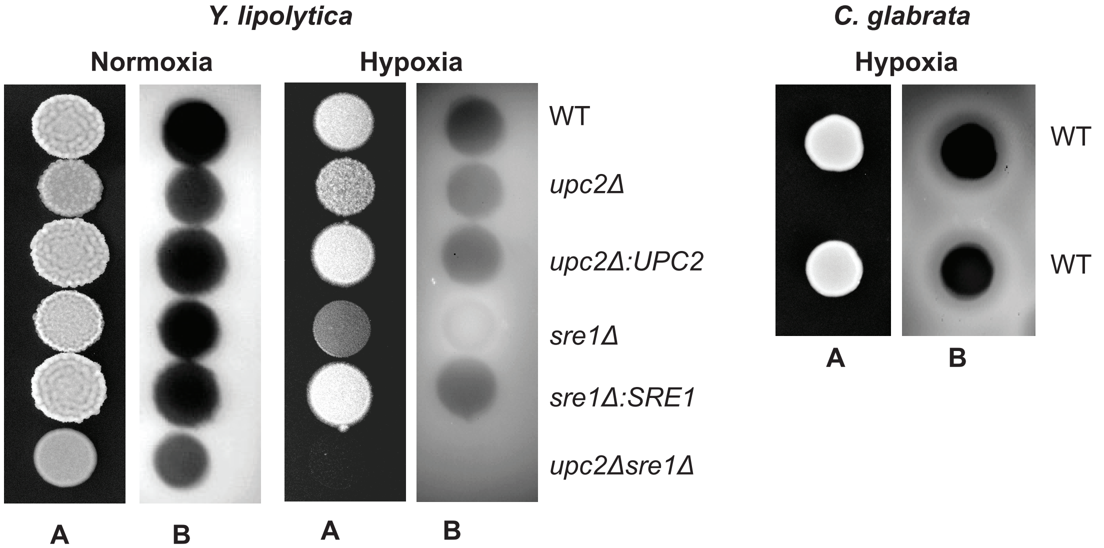
Y. lipolytica
Normoxia
Hypoxia
WT
upc2Δ
upc2Δ:UPC2
sre1Δ
sre1Δ:SRE1
upc2Δsre1Δ
A
B
A
B
C. glabrata
Hypoxia
WT
WT
A
B
