# Supplementary material for: Zinc Finger Transcription Factors Displaced SREBP Proteins as the Major Sterol Regulators during Saccharomycotina Evolution
Source: PLoS Genet. 2014 Jan 16;10(1):e1004076. doi: 10.1371/journal.pgen.1004076 (PMC3894159; doi:10.1371/journal.pgen.1004076)
Supplement: Table S1 — Strains used. (DOCX) [file pgen.1004076.s008.docx]

**Table S1: Strains used.**

| **Strain** | **Genotype** | **Source/Reference** |
| --- | --- | --- |
| *Y. lipolytica* Po1d | Mat A, *leu2–270, ura3–302, xpr2–322* | (Barth and Gaillardin, 1996) |
| *Y. lipolytica*  JMY330 | Mat A, *leu2–270, ura3–302::URA3, xpr2–322* | (Beopoulos et al., 2008) |
| *Y. lipolytica*  JMY2900 | Mat A, *leu2–270::LEU2, xpr2–322* | This study, derived from JMY303 |
| *Y. lipolytica*  SMY1 | Mat A, *leu2–270, ura3–302, xpr2–322, upc2Δ::URA3* | This study, derived from Po1d |
| *Y. lipolytica*  SMY2 | Mat A, *leu2–270::LEU2, ura3–302, xpr2–322, upc2Δ::URA3* | This study, derived from SMY1 |
| *Y. lipolytica*  SMY3 | Mat A, *leu2–270, ura3–302, xpr2–322, sre1Δ::LEU2* | This study, derived from Po1d |
| *Y. lipolytica*  SMY4 | Mat A, *leu2–270, ura3–302, xpr2–322, sre1Δ::LEU2, upc2Δ::URA3* | This study, derived from SMY3 |
| *Y. lipolytica*  SMY5 | Mat A, *leu2–270, ura3–302::URA3, xpr2–322, sre1::LEU2* | This study, derived from JMY330 |
| *Y. lipolytica*  SMY6 | Mat A, *leu2–270::LEU2, ura3–302, xpr2–322, upc2Δ::URA3, HYG-pUPC2* | This study, derived from SMY2 |
| *Y. lipolytica*  SMY7 | Mat A, *leu2–270, ura3–302::URA3, xpr2–322, sre1::LEU2, HYG-pSRE1* | This study, derived from SMY5 |
| *Y. lipolytica*  SMY8 | Mat A, *leu2–270, ura3–302::URA3, xpr2–322, sre1Δ::LEU2* | This study, derived from SMY3 |
| *E. coli* JMP802 | JMP62-LEU2ex, pPOX2 expression vector with excisable LEU2ex marker | (Haddouche et al., 2010) |
| *E. coli* JMP803 | JMP62-URA3ex, pPOX2 expression vector with excisable URA3ex marker | (Haddouche et al., 2011) |
| *E. coli* JMP804 | JMP62-*HygEx* pPOX2 expression vector with excisable *HygEx* marker | (Nicaud et al., 2011) |
| *E. coli* SMP1 | JMP62-*HygEx*-pUPC2 | This study, derived from JMP804 |
| *E. coli* SMP2 | JMP62-*HygEx*-pSRE1 | This study, derived from JMP804 |
