# Supplementary material for: Zinc Finger Transcription Factors Displaced SREBP Proteins as the Major Sterol Regulators during Saccharomycotina Evolution
Source: PLoS Genet. 2014 Jan 16;10(1):e1004076. doi: 10.1371/journal.pgen.1004076 (PMC3894159; doi:10.1371/journal.pgen.1004076)
Supplement: Table S4 — Oligonucleotide primer sequences. (DOCX) [file pgen.1004076.s011.docx]

**Table S4: Oligonucleotide Primer Sequences**

| **Primer Name** | **Primer Sequence** | **Purpose** |
| --- | --- | --- |
| UPC2_p1 | GAGGACCGACCTTGCATAAA | *UPC2* disruption cassette |
| UPC2_p2_uraa | **atggctctctgggcggaattc**GATTCCACTCTCAAGACATG | *UPC2* disruption cassette |
| UPC2_t1_urab | **gttgttgtgtttctcggaattc**TAGTACATAGTCAACGTAAC | *UPC2* disruption cassette |
| UPC2_t2 | TAGAGGCTCCATTGGTGGTC | *UPC2* disruption cassette |
| URA-A | GAATTCCGCCCAGAGAGCCATTGACGTTC | *UPC2* disruption cassette |
| URA-B | GAATTCCGAGAAACACAACAACATGCCCC | *UPC2* disruption cassette |
| SRE1_p1 | CAGATAGTTGGGTGGGCAGT | *SRE1* disruption cassette |
| SRE1_p2_leua | **atgatgactcaggcggaattc**TTGGTTGCGTGTAGTGTTGT | *SRE1* disruption cassette |
| SRE1_p2_uraa | **atggctctctgggcggaattc**TTGGTTGCGTGTAGTGTTGT | *SRE1* disruption cassette |
| SRE1_t1_leub | **cttgttactgtatattcgaattc**GTAGCTGAGGCTCGAATGGG | *SRE1* disruption cassette |
| SRE1_t1_urab | **gttgttgtgtttctcggaattc**GTAGCTGAGGCTCGAATGGG | *SRE1* disruption cassette |
| SRE1_t2 | ATGTAGAGGGCTCTCGTTGC | *SRE1* disruption cassette |
| LEU-A | GAATTCCGCCTGAGTCATCATTTATTTACC | *SRE1* disruption cassette |
| LEU-B | GAATTCGAATATACAGTAACAAGCTACCAC | *SRE1* disruption cassette |
| UPC2_in_F | CTTGGGACTGGAATGTTCGT | *UPC2* disruption confirmation (internal) |
| UPC2_in_R | CCACCGTCATGTAGTTGTGC | *UPC2* disruption confirmation (internal) |
| UPC2_out_F | CTCGGCTCGAGGTATCATTT | *UPC2* disruption confirmation (external) |
| UPC2_out_R | CCAGGAAGGTCTGGTACAGC | *UPC2* disruption confirmation (external) |
| SRE1_in_F | GCAAAGTCATGAAGCCCAAG | *SRE1* disruption confirmation (internal) |
| SRE1_in_R | TCAGCACAGTGGCCTTATTG | *SRE1* disruption confirmation (internal) |
| SRE1_out_F | AGAGGCTGCACTTTCCTCAA | *SRE1* disruption confirmation (external) |
| SRE1_out_R | CCGAACGGCAGTGTTTACTT | *SRE1* disruption confirmation (external) |
| URI_xhoI_F | ggcgccCTCGAGAGCGTAGACTCAGGCTCAGG | *UPC2* reintegration construct |
| URI_avrII_R | ggcgccCCTAGGGTTACGTTGACTATGTACTA | *UPC2* reintegration construct |
| SRI_claI_F | ggcgccATCGATACCGAAAATATGAGAACCCT | *SRE1* reintegration construct |
| SRI_bamHI_R | ggcgccGGATCCCCCATTCGAGCCTCAGCTAC | *SRE1* reintegration construct |
| SR_P5_i6 | ACACTCTTTCCCTACACGACGCTCTTCCGATCTagcta***T** | RNA-seq Adaptor |
| SR_P7_i6 | tagctAGATCGGAAGAGCTCGTATGCCGTCTTATGCTTG | RNA-seq Adaptor |
| SR_P5_i10 | ACACTCTTTCCCTACACGACGCTCTTCCGATCTcgatc***T** | RNA-seq Adaptor |
| SR_P7_i10 | gatcgAGATCGGAAGAGCTCGTATGCCGTCTTATGCTTG | RNA-seq Adaptor |
| SR_P5_i11 | ACACTCTTTCCCTACACGACGCTCTTCCGATCTgctag***T** | RNA-seq Adaptor |
| SR_P7_i11 | ctagcAGATCGGAAGAGCTCGTATGCCGTCTTATGCTTG | RNA-seq Adaptor |
| yl_erg3_rt_f | TTTGGAGCGCTTCTGTACCT | qPCR primer for *ERG3* |
| yl_erg3_rt_r | CTGACTTCTGCGACAAACCA | qPCR primer for *ERG3* |
| yl_erg25_rt_f | ACAAGCACATCCACAAGCAG | qPCR primer for *ERG25* |
| yl_erg25_rt_r | ATCCACACAGACACGGTGAA | qPCR primer for *ERG25* |
| yl_erg2_rt_f | ACGACCGATATGGCAAGTTC | qPCR primer for *ERG2* |
| yl_erg2_rt_r | GAAGTGGTCATCGGCGTAAT | qPCR primer for *ERG2* |
| yl_erg2_2_rt_f | CATCTCGCTGCTCGTGTTAG | qPCR primer for *ERG2_2* |
| yl_erg2_2_rt_r | TCATGATTTCGGTGGCATTA | qPCR primer for *ERG2_2* |
| yl_erg11_rt_f | CCCAGCAAAAGATTCGTGAT | qPCR primer for *ERG11* |
| yl_erg11_rt_r | CTGGGGAGTCATTCGCTTAC | qPCR primer for *ERG11* |
| yl_act1_rt_f | TCCAGGCCGTCCTCTCCC | qPCR primer for *ACT1* |
| yl_act1_rt_r | GGCCAGCCATATCGAGTCGCA | qPCR primer for *ACT1* |

***T** indicates phosphorothioate bond, for stabilisation and resistance to nuclease

lowercase bold sequence indicates complementarity to either LEUA, LEUB, URAA or URAB

uppercase underlined sequence indicates restriction site contained within primer.
