# Supplementary material for: Zinc Finger Transcription Factors Displaced SREBP Proteins as the Major Sterol Regulators during Saccharomycotina Evolution
Source: PLoS Genet. 2014 Jan 16;10(1):e1004076. doi: 10.1371/journal.pgen.1004076 (PMC3894159; doi:10.1371/journal.pgen.1004076)
Supplement: Table S5 — RNA-seq libraries. (DOCX) [file pgen.1004076.s012.docx]

**Table S5 – RNA-seq libraries.**

| **Genotype** | **Stain/Replicate** | **Growth Condition** | **Total Mapped Reads** |
| --- | --- | --- | --- |
| Wildtype | JMY2900 Rep 1 | normoxia1 | 5,962,848 |
| Wildtype | JMY2900 Rep 2 | normoxia2 | 6,520,352 |
| Wildtype | JMY2900 Rep 3 | normoxia3* | 25,295,430 |
| Wildtype | JMY2900 Rep 4 | normoxia4* | 26,056,657 |
| *sre1Δ* | SMY5 Rep 1 | normoxia1 | 9,367,826 |
| *sre1Δ* | SMY5 Rep 2 | normoxia2 | 5,009,029 |
| *sre1Δ* | SMY8 Rep 1 | normoxia3 | 6,724,511 |
| *upc2Δ* | SMY2 Rep 1 | normoxia1 | 5,162,364 |
| *upc2Δ* | SMY2 Rep 2 | normoxia2 | 7,300,149 |
| *upc2Δ* | SMY2 Rep 3 | normoxia3 | 8,572,280 |
| *sre1Δ/upc2Δ* | SMY4 Rep 1 | normoxia1 | 10,305,535 |
| *sre1Δ/upc2Δ* | SMY4 Rep 2 | normoxia2 | 8,209,011 |
| Wildtype | JMY2900 Rep 1 | hypoxia1 | 5,783,571 |
| Wildtype | JMY2900 Rep 2 | hypoxia2 | 5,879,118 |
| Wildtype | JMY2900 Rep 3 | hypoxia3 | 5,903,250 |
| Wildtype | JMY2900 Rep 4 | hypoxia4* | 24,561,187 |
| Wildtype | JMY2900 Rep 5 | hypoxia5* | 22,567,145 |
| *sre1Δ* | SMY8 Rep 1 | hypoxia1 | 6,614,038 |
| *sre1Δ* | SMY5 Rep 1 | hypoxia2 | 5,506,328 |
| *upc2Δ* | SMY4 Rep 1 | hypoxia1 | 8,041,298 |
| *upc2Δ* | SMY4 Rep 2 | hypoxia2 | 5,480,571 |
| *upc2Δ* | SMY4 Rep 3 | hypoxia3 | 6,822,649 |

*libraries generated and sequences by GATC using an Illumina Hiseq 2500
